# Post-translational deregulation of YAP1 is genetically controlled in rat liver cancer and determines the fate and stem-like behavior of the human disease

**DOI:** 10.18632/oncotarget.10246

**Published:** 2016-06-23

**Authors:** Maria M. Simile, Gavinella Latte, Maria I. Demartis, Stefania Brozzetti, Diego F. Calvisi, Alberto Porcu, Claudio F. Feo, Maria A. Seddaiu, Lucia Daino, Carmen Berasain, Maria L. Tomasi, Matias A. Avila, Francesco Feo, Rosa M. Pascale

**Affiliations:** ^1^ Department of Clinical and Experimental Medicine, Division of Experimental Pathology and Oncology, University of Sassari, Sassari, Italy; ^2^ Department of Surgery “Pietro Valdoni”, University of Rome ‘Sapienza’', Rome, Italy; ^3^ Department of Clinical and Experimental Medicine, Division of Surgery, University of Sassari, Sassari, Italy; ^4^ Division of Hepatology, Centro de Investigación Médica Aplicada (CIMA), University of Navarra, Pamplona, Spain; ^5^ CIBERehd, Instituto de Salud Carlos III, Madrid, Spain; ^6^ IDISNA, Navarra Institute for Health Research, Pamplona, Spain; ^7^ Division of Gastroenterology, Cedars-Sinai Medical Center, Los Angeles, CA, USA; ^8^ USC Research Center for Liver Diseases, Keck School of Medicine of University of Southern California, Los Angeles, CA, USA

**Keywords:** hepatocarcinogenesis, stem cells, progression, gene expression profile, yap targets

## Abstract

Previous studies showed that YAP1 is over-expressed in hepatocellular carcinoma (HCC). Here we observed higher expression of Yap1/Ctgf axis in dysplastic nodules and HCC chemically-induced in F344 rats, genetically susceptible to hepatocarcinogenesis, than in lesions induced in resistant BN rats. In BN rats, highest increase in Yap1-tyr357, p73 phosphorylation and Caspase 3 cleavage occurred. In human HCCs with poorer prognosis (< 3 years survival after partial liver resection, HCCP), levels of YAP1, CTGF, 14–3–3, and TEAD proteins, and YAP1-14-3-3 and YAP1-TEAD complexes were higher than in HCCs with better outcome (> 3 years survival; HCCB). In the latter, higher levels of phosphorylated YAP1-ser127, YAP1-tyr357 and p73, YAP1 ubiquitination, and Caspase 3 cleavage occurred. Expression of stemness markers NANOG, OCT-3/4, and CD133 were highest in HCCP and correlated with YAP1 and YAP1-TEAD levels. In HepG2, Huh7, and Hep3B cells, forced YAP1 over-expression led to stem cell markers expression and increased cell viability, whereas inhibition of YAP1 expression by specific siRNA, or transfection of mutant YAP1 which does not bind to TEAD, induced opposite alterations. These changes were associated, in Huh7 cells transfected with YAP1 or YAP1 siRNA, with stimulation or inhibition of cell migration and invasivity, respectively. Furthermore, transcriptome analysis showed that YAP1 transfection in Huh7 cells induces over-expression of genes involved in tumor stemness. In conclusion, Yap1 post-translational modifications favoring its ubiquitination and apoptosis characterize HCC with better prognosis, whereas conditions favoring the formation of YAP1-TEAD complexes are associated with aggressiveness and acquisition of stemness features by HCC cells.

## INTRODUCTION

Hepatocellular carcinoma (HCC) is a frequent and fatal human cancer [1, 2]. Previous work in our laboratory, has shown highest over-expression of iNOS/NF-kB (inducible nitric oxide synthase/nuclear factor-kB), RAS/ERK (extracellular signal-regulated kinase), FoxM1 (Forkhead box M1B), Mybl2 (v-Myb avian myeloblastosis viral oncogene homolog-like2), and decrease in methionine metabolism, in the human HCC subtype with poorer prognosis (< 3 years survival, after partial liver resection, HCCP), and low/absent in the human HCC subtype characterized by better outcome (> 3 years survival; HCCB) [1]. Interestingly, utmost upregulation of above signaling pathways in rapidly progressing HCC chemically induced in Fisher 344 (F344) rats, genetically susceptible to hepatocarcinogenesis, contrasts with low/absent deregulation of the same cascades in slowly progressing lesions of genetically resistant Brown Norway (BN) rats, suggesting a genetic control of signaling deregulation and HCC aggressivity [2].

**Figure 1:**
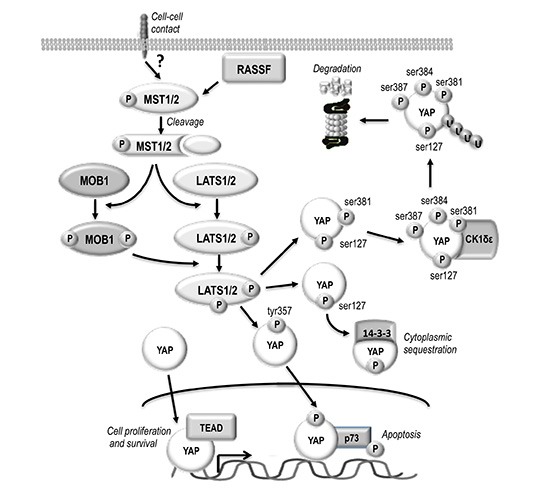
Schematic representation of HIPPO/YAP pathway The upstream activators of MST1/2 are not defined although cell–cell contact probably is an important stimulus. RASSF family proteins could link MST1/2 to extracellular signals facilitating their activation before the proteolytic cleavage. Indeed, most catalytically active MST1/2 in the liver are in a truncated form that lacks the autoregulatory carboxy-terminus. Mst1/2 are required to phosphorylate MOB1. Phospho-MOB1 is likely to facilitate the activation of an intermediary kinase, which phosphorylates YAP. YAP phosphorylation at ser127 allows 14-3-3 binding and cytoplasmic sequestration. Subsequent phosphorylation of YAP-ser127 at ser381 by LATS1/2 followed by phosphorylation at ser384 and ser387 by CK1δε allows ubiquitinylation and degradation. The phosphorylation of YAP at tyr357 promotes nuclear translocation and binding to the oncosuppressor p73. Nuclear migration of not phosphorylated YAP protein is followed by the formation of YAP/TEAD complex that enhances cell proliferation and inhibits apoptosis.

Recent studies indicate the deregulation of Hippo/YAP signaling in oval cells and HCC [1, 3, 4]. A complex network of not yet completely known mechanisms regulates this pathway. Some inputs are associated with plasma membrane and might transmit information from the extracellular milieu or cell-cell contacts [1, 5]. MST1/2 (homologues of Hpo), MOB1A/B (preimplantation protein 1, mouse, homolog of), and LATS1/2 (Wts homologues) are involved in YAP (Yes kinase-associated protein) phosphorylation (Figure 1). YAP1 phosphorylation at ser127 residue allows 14-3-3 binding and cytoplasmic sequestration and inactivation, whereas phosphorylation at tyr357 promotes YAP1 nuclear translocation and binding to the oncosuppressor p73 [1, 5]. Moreover, phosphorylation at ser381 by LATS1/2 primes YAP for subsequent phosphorylation at ser384 and ser387, presumably by casein kinase-1 (CK1δ/ε), allowing the recruitment to YAP of E3 ubiquitin ligase SCFβ-TRCP (Skp1-Cullin1-F-box protein beta-transducin repeat-containing protein) followed by YAP ubiquitination and proteolysis [5]. Finally, nuclear migration of not phosphorylated YAP protein is followed by the formation of YAP/TEAD complex that enhances cell proliferation and inhibits apoptosis [1, 5].

Cumulating observations indicate that the Hippo/YAP signaling is critical for HCC development [6, 7]. Connective tissue growth factor (CTGF) expression can be stimulated through the Amphiregulin (AREG)-(Epidermal growth factor receptor) EGFR-cascade in a crosstalk with YAP in HCC cells [7]. YAP inhibition is able to restore hepatocyte differentiation in advanced HCC [8]. Furthermore, recent results provide immunohistochemical evidence of Yap overexpression in carcinogen-induced early liver preneoplastic lesions and HCC of rats and human HCC [9]. Verteporfin, an inhibitor of Yap-Tead complex, reduced oval cell proliferation and preneoplastic and neoplastic lesions development in the rat liver [9]. In accordance with the latter findings, activation of YAP due to the loss of the YAP inhibitor, WW45, leads to unrestrained hepatic oval cell proliferation and oval cell-driven tumor development in mice [10]. The latter findings envisage a critical role of the Hippo/YAP signaling in stem cell proliferation/expansion. Moreover, YAP overexpression occurs most often in HCCs and combined Cholangiocarcinoma-HCCs displaying stemness markers [11]. Importantly, recent observations suggest the implication of stem cells in HCC aggressiveness and recurrence [12].

Although the aforementioned data convincingly indicate that YAP is oncogenic in liver cancer, other findings support an oncosuppressor effect of YAP in cancer. Indeed, YAP is able to inhibit the growth of human malignant cells by activating apoptogenic p73 pathway [13], the PML (Promyelomonocytic leukemia) oncosuppressor protein interacts with YAP1, enhancing its stabilization [14], and YAP increases chemosensitivity of HCC cells by modulating p53 [15]. YAP also suppresses head and neck [16] and breast [17] cancers.

Recent findings showed that TAZ (Transcriptional co-activator with PDZ-binding motif), another downstream effector of the Hippo signaling pathway, but not YAP, is predominantly expressed in HCC. In the same sample collection, nonetheless, YAP overexpression was associated with HCC poor prognosis, and a compensatory YAP upregulation following TAZ depletion conferred cancer stem cell-like properties to HCC cells [18].

We hypothesized that variations of YAP1 role in HCC growth and stemness might depend on differences in its post-translational deregulation. We tested this hypothesis by analyzing the relationship of post-translational deregulation of YAP1, in prognostic subtypes of HCC, with the genetic predisposition to the disease and the activity of stemness key genes. We also analyzed the mechanism involved in the upregulation of these genes by YAP1.

## RESULTS

### YAP/CTGF pathway is under genetic control in rat HCC

**Figure 2:**
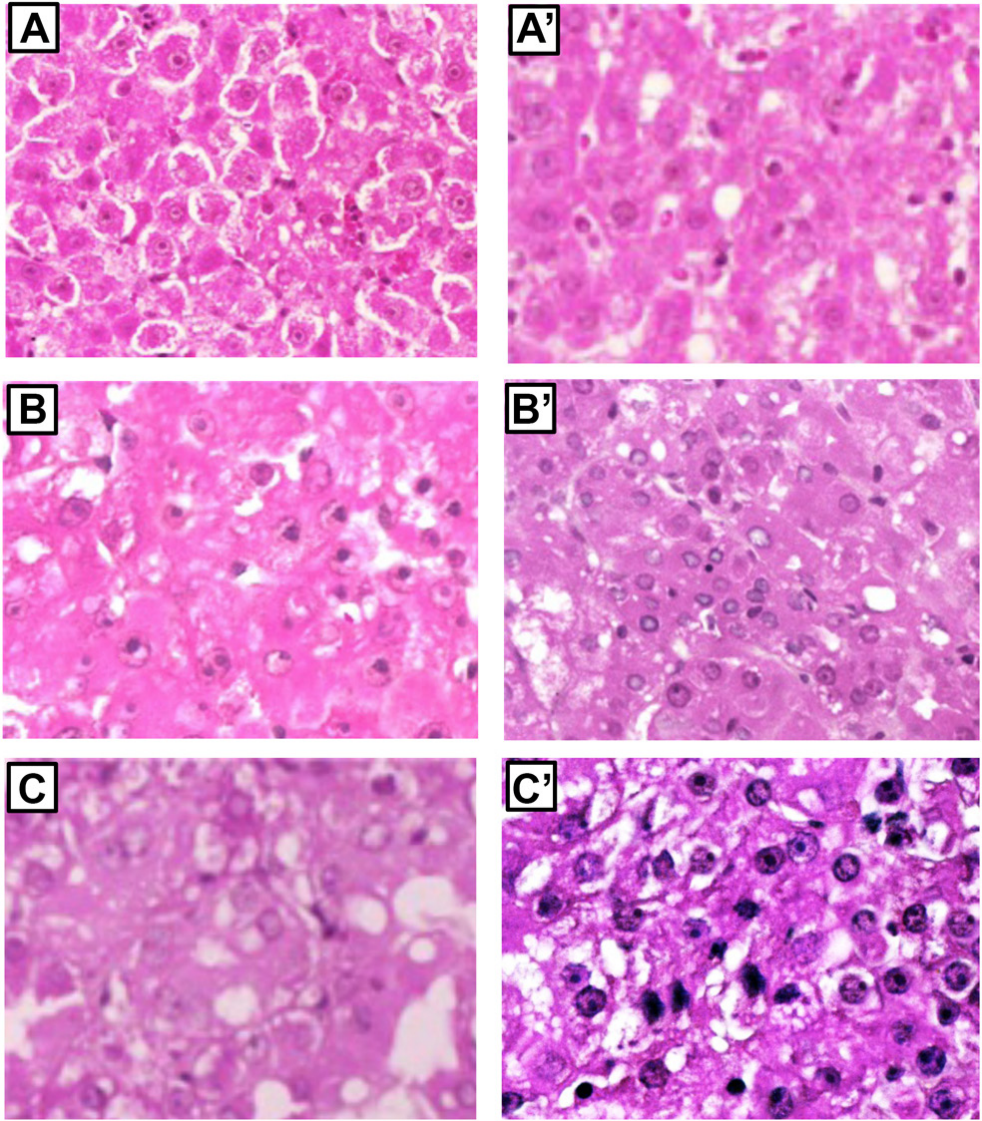
Preneoplastic and neoplastic liver lesion of F344 and BN rats (**A**) low-grade dysplastic nodule of BN rats. (**B** and **C**) high-grade dysplastic nodules of F344 rats. (**A'**) well-differentiated HCC of BN rats. (**B'** and **C'**) moderately differentiated and poorly differentiated HCC, respectively, of F344 rats. Low-grade dysplastic nodules were constituted prevalently by eosinophilic hepatocytes. High-grade nodules exhibited prevalently small hepatocytes with high nuclear:cytoplasmic ratio, hepatocytes in nests or pseudo-gland formation, and cytoplasmic basophilia. Initial magnification: A–C, A', B', 200×; C', 400×.

Six weeks after initiation, foci of altered hepatocytes (mostly clear/eosinophilic cell lesions) occupied 76– 82% of the liver in F344 and BN rats. At 15–32 weeks, relatively few nodules were present in BN rat liver and nodule volume was much lower in BN than F344 rats (cm^3^: 0.032–0.11 and 0.11–0.52 in BN and F344 rats, respectively). Histologic analysis of pooled nodules showed that at 15 weeks all dysplastic nodules of both rat strains were low-grade lesions, whereas at 32 weeks low-grade and high-grade dysplastic nodules were present in BN and F344 rats, respectively (Figure 2). Poorly differentiated and moderately differentiated HCCs developed at 57 weeks in F344 rats, whereas 90% well differentiated HCC developed at 60 weeks in BN rats (Figure 2).

**Figure 3:**
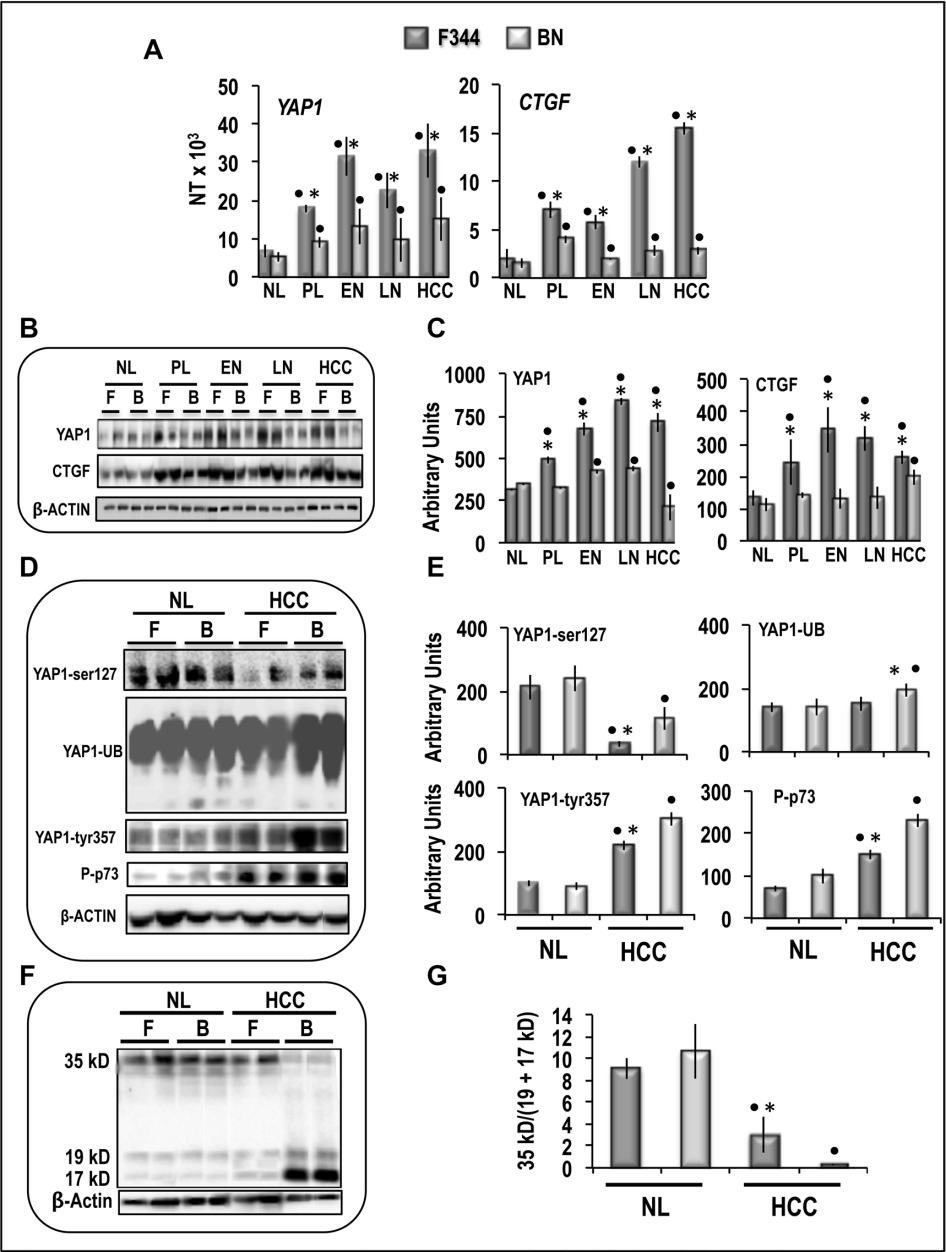
Expression and post-translational modifications of YAP1 in preneoplastic and neoplastic rat liver lesions induced in rats by “resistant hepatocyte” protocol (**A**) mRNA levels of *Yap* and *Ctgf*, determined by QRT-PC, in normal liver (NL), pre-neoplastic liver (4–6 weeks after initiation, PL), early nodules (15 weeks, EN), late nodules (32 weeks, LN), and HCCs. N Target (NT) = 2^-ΔCt^; ΔCt = Ct RNR18S-Ct target gene. (**B**) Representative Western blots of Yap1 and Ctgf proteins. (**C**) Chemiluminescence analysis: optical densities were normalized to β-actin levels and expressed in arbitrary units. (**D**) Representative Western blots of YAP phosphorylated at ser127 and tyr357, YAP ubiquitinated, and phosphorylated p73. (**E**) Chemiluminescence analysis: optical densities were normalized to β-actin levels and expressed in arbitrary units. Data are means (SD) of 4–10 rats. Tukey-Kramer test: Point, different from NL for at least *P* < 0.02. Asterisk, F344 different from BN for at least *P* < 0.01. (**F**) Representative Western blot and (**G**) Chemiluminescence analysis showing means (SD) of 5 experiments of Caspase 3 cleavage. in NL and HCC of F344 (F) and BN (B) rats. Optical densities of the peaks were normalized to β-actin levels and expressed in arbitrary units. Tukey-Kramer test: Point, different from NL for at least *P* < 0.0001. Asterisk, F344 different from BN for at least *P* < 0.0001.

Quantitative RT-PCR (QRT-PCR) analysis showed the absence of differences of *Yap1* and *Ctgf* expression between normal livers from F344 and BN rats, whereas mRNAs levels of both genes were significantly higher in preneoplastic liver, dysplastic nodules and HCCs of F344 than BN rats (Figure 3A). Yap1 and Ctgf protein levels sharply increased in F344 rat lesions, compared to normal liver, whereas no changes/lower increase occurred in BN rats (Figure 3B, 3C). These findings were associated with a sharp decrease in phosphorylated Yap1-ser127 and increase in phosphorylated Yap1-tyr357 in HCC of both rat strains, with respect to normal liver, with lowest values of pYap1-ser127 in F344 HCC and highest values of pYap1-tyr357 in BN HCC, respectively (Figure 3D, 3E). A significant rise of Yap1 ubiquitination occurred only in BN HCC, and p73 phosphorylation increased in HCC of both strains, with highest values in BN rats (Figure 3D, 3E). Accordingly, HCC of both strains exhibited a significant increase in Caspase 3 cleavage [expressed as fall in the 36/(19+17) kD ratio], taken as a measure of apoptosis, compared to normal liver, which was 4-fold higher in BN than F344 rats (Figure 3F, 3G).

### YAP1 post-translational changes in human HCC prognostic subgroups

Above results suggest a link between the genetic predisposition to HCC and the deregulation of YAP1 signaling, and envisage a role of the latter in HCC aggressiveness. This prompted us to analyze YAP1 expression and post-translational regulation in human HCCs with different growth rate and propensity to progress. Two groups of 20 patients with HCCB and HCCP were used for these experiments (Table 1). No significant differences between the two groups occurred as concerns patients' sex, etiology, presence of cirrhotic liver, and Edmondson-Steiner grade. Significantly higher tumor size, alpha-fetoprotein secretion, proliferation index (Ki67 expression), YAP1 expression, and Midkine expression (as index of poor differentiation), [19, 20], were found in HCCP than in HCCB.

**Table 1:** Clinicopathological features of HCC patients

	HCCB	HCCP
No. of patients Male Female	11 9	13 7
Age (Mean ± SD)	63.4 ± 12.8	67.2 ± 8.9
Etiology HBV HCV Ethanol	14 4 2	17 3 0
Cirrhosis + –	18 2	17 3
Tumor size^a^ > 5 cm < 5 cm	7 13	15 5
Edmondson and Steiner grade I II III IV	1 12 7 0	0 8 10 2
Alpha-fetoprotein secretion^b^ > 300 ng/ml of serum < 300 ng/ml of serum	8 12	17 3
Proliferation index (×10^3^)^c^	7.2 ± 1.3	14.9 ± 2.7
YAP1 expression (× 10^3^)^d^	2.8 ± 0.9	5.8 ± 1.2
Midkine expression (× 10^3^)^e^	1.1 ± 1.0	47.9 ± 34.8
Survival after partial liver resection (months). Mean ± SD^f^	54 ± 12.2	22 ± 8.3

HCCB, HCC with better prognosis (survival > 3 years); HCCP,

HCC with poorer prognosis (survival < 3 years).

a Fisher Exact Test. *p* = 0.025.

b Fisher Exact Test: *p* = 0.008.

c Ki67 expression (quantitative RT-PCR. 2-ΔCt; ΔCt = Ct RNR18-Ct target gene): *p* < 0.0001.

d QRT-PCR. 2-ΔCt; ΔCt = Ct RNR18-Ct target gene: *p* < 0.001.

e QRT-PCR. 2-ΔCt; ΔCt = Ct RNR18-Ct target gene: *p* < 0.0005.

f *p* < 0.0001.

**Figure 4:**
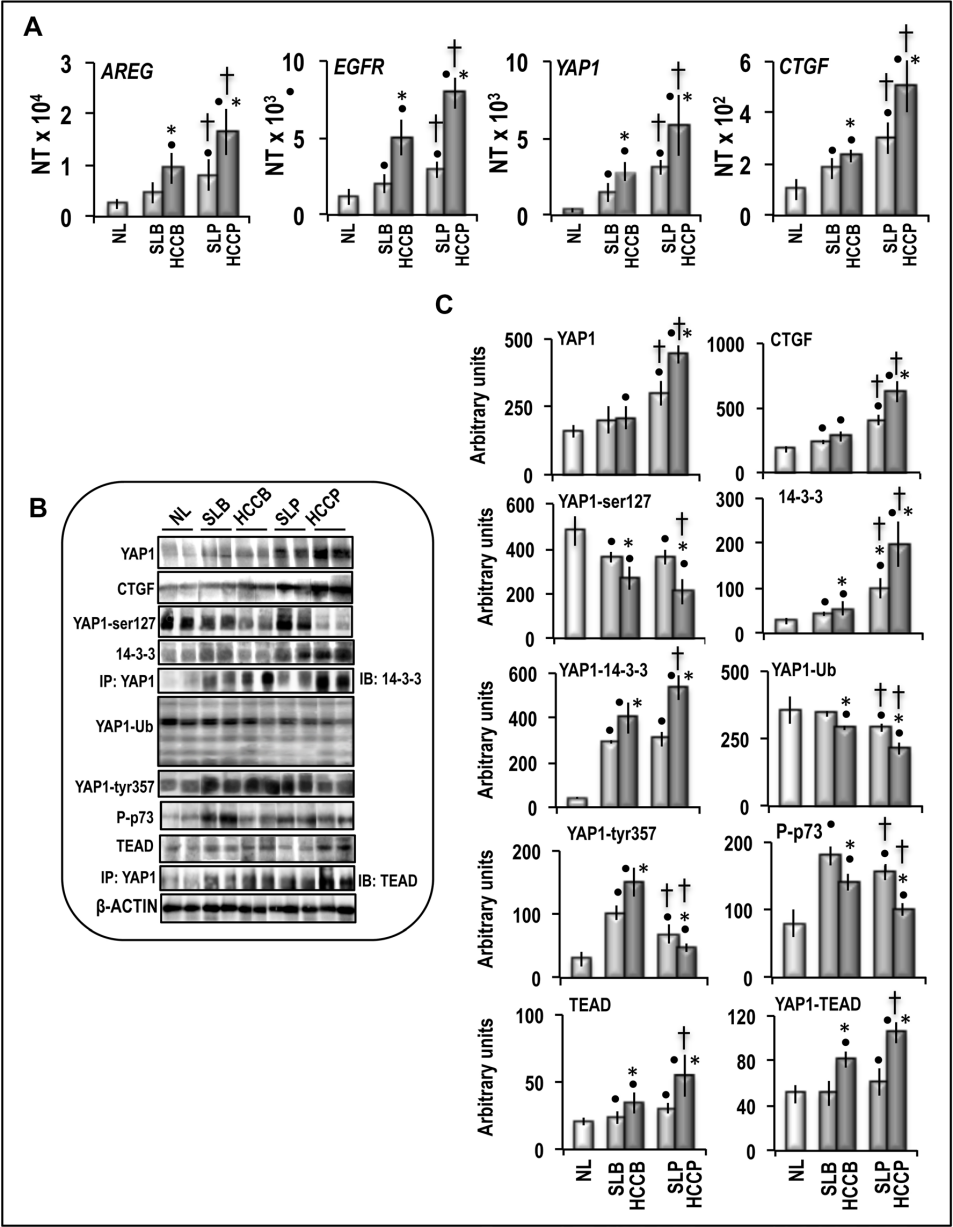
Expression of AREG/EGFR/YAP/CTGF pathway and post-translational modifications of YAP1 in human normal liver (NL), HCC, and corresponding surrounding liver (SL) HCCs were divided in two distinct categories based on the length of patient survival after partial liver resection: HCC with better outcome (survival > 3 years; HCCB), and HCC with poorer outcome (survival < 3 years; HCCP). (**A**) mRNA levels *of AREG, EGFR, YAP1*, and *CTGF* genes were determined by QRT-PCR. N Target (NT) = 2-ΔCt; ΔCt = Ct RNR18-Ct target gene. Data are means (SD) of NT of 5 normal livers (NL) and 20 of each HCC subtypes and corresponding SL. Mann-Whitney test: Point, different from NL for *P* < 0.001. Asterisk, different from SL for at least *P* < 0.01. Dagger, HCCP/SLP different from HCCB/SLB for *P* < 0.001. (**B**) Representative Western blots of Yap1, CTGF and phosporylated and ubiquitinylated YAP1, phosphorylated p73, 14-3-3, TEAD, and YAP1-14-3-3 and YAP1-TEAD complexes in NL, HCCB, HCCP and corresponding SL. (**C**) Chemiluminescence analysis: optical densities were normalized to β-actin levels and expressed in arbitrary units. Data are means (SD) of 5–20 experiments. Mann-Whitney test: Point, different from NL for at least *P* < 0.01. Asterisk, different from SL for at least *P* < 0.01. Dagger, HCCP/SLP different from HCCB/SLB for at least *P* < 0.01.

**Figure 5:**
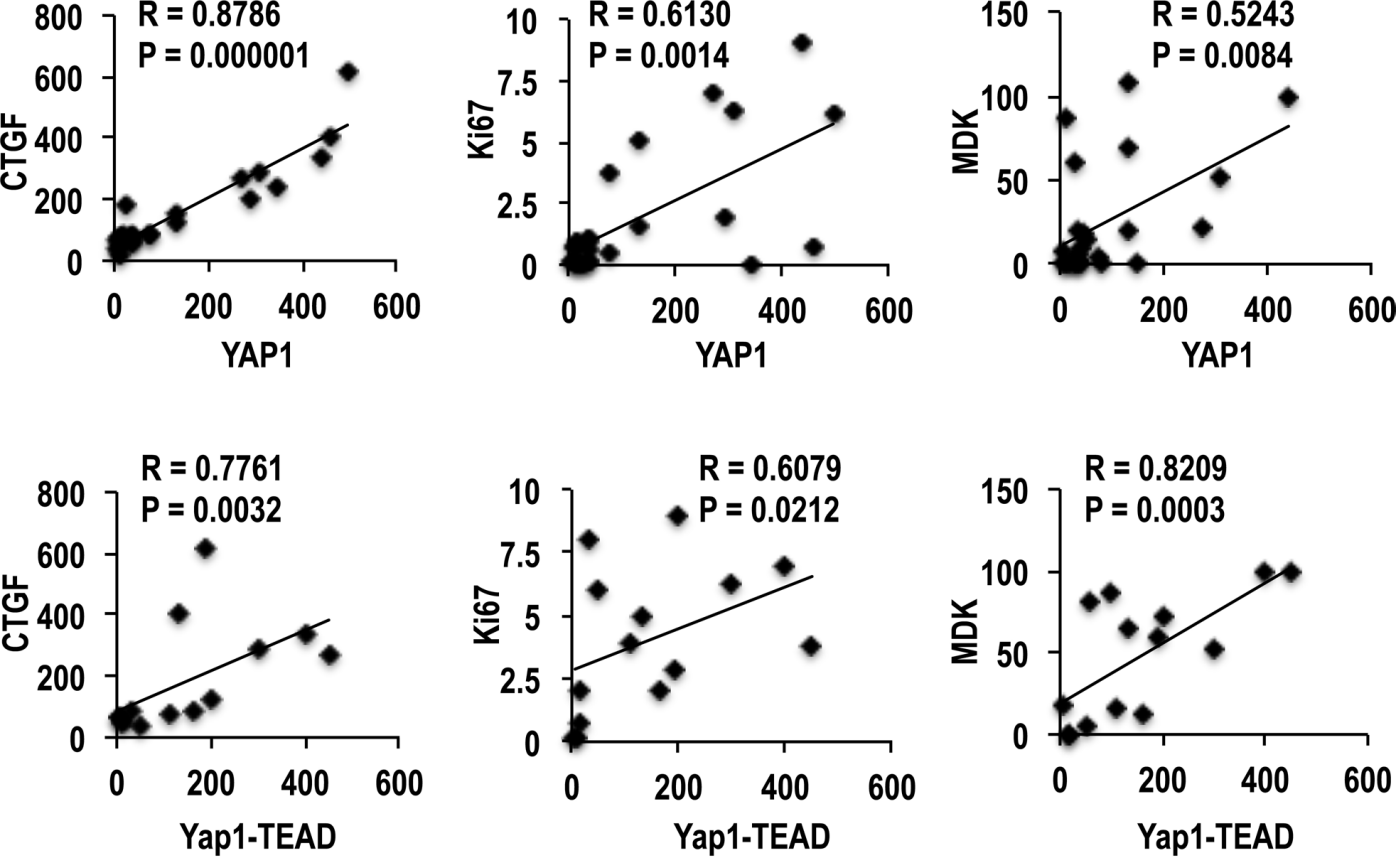
Correlation of YAP1 and YAP1-TEAD complex with CTGF, Ki67, and MDK (Midkine) in human HCCs A total of 18 cases (9 HCCB and 9 HCCP) were used for the Spearman's correlation analysis of YAP1, and a total of 15 cases (7 HCCB and 8 HCCP) were used for the correlation analysis of YAP1-TEAD complex.

The evaluation of the AREG/EGFR/YAP/CTGF axis in human HCC subgroups showed an increase of *AREG, EGFR, YAP* and *CTGF* mRNA expression, with respect to normal liver, which was progressively higher from SLB, HCCB, SLP to HCCP (Figure 4A). Western blot analysis (Figure 4B, 4C) showed progressive increase of YAP1 and CTGF proteins from HCCB to SLP, and HCCP. Phosphorylated YAP1-ser127 decreased in SL and HCC, with lowest values in HCCP. However, 14-3-3 protein and YAP1-14-3-3 complex increased in SL and HCC, with highest values in HCCP. YAP1 ubiquitinylation progressively decreased from HCCB to SLP and HCCP, lowest values were observed in HCCP. TEAD protein and YAP1-TEAD complex showed small increase/no change in SLs and highest increase in HCCP (Figure 4B, 4C). These results indicate that YAP1 post-translational regulation supports HCC progression by favoring the formation of YAP1-TEAD complex. The relationships between YAP1 protein and YAP1-TEAD complex levels and HCC progression were confirmed by their significant correlation with CTGF, Ki67, and MDK protein expression (Figure 5).

**Figure 6:**
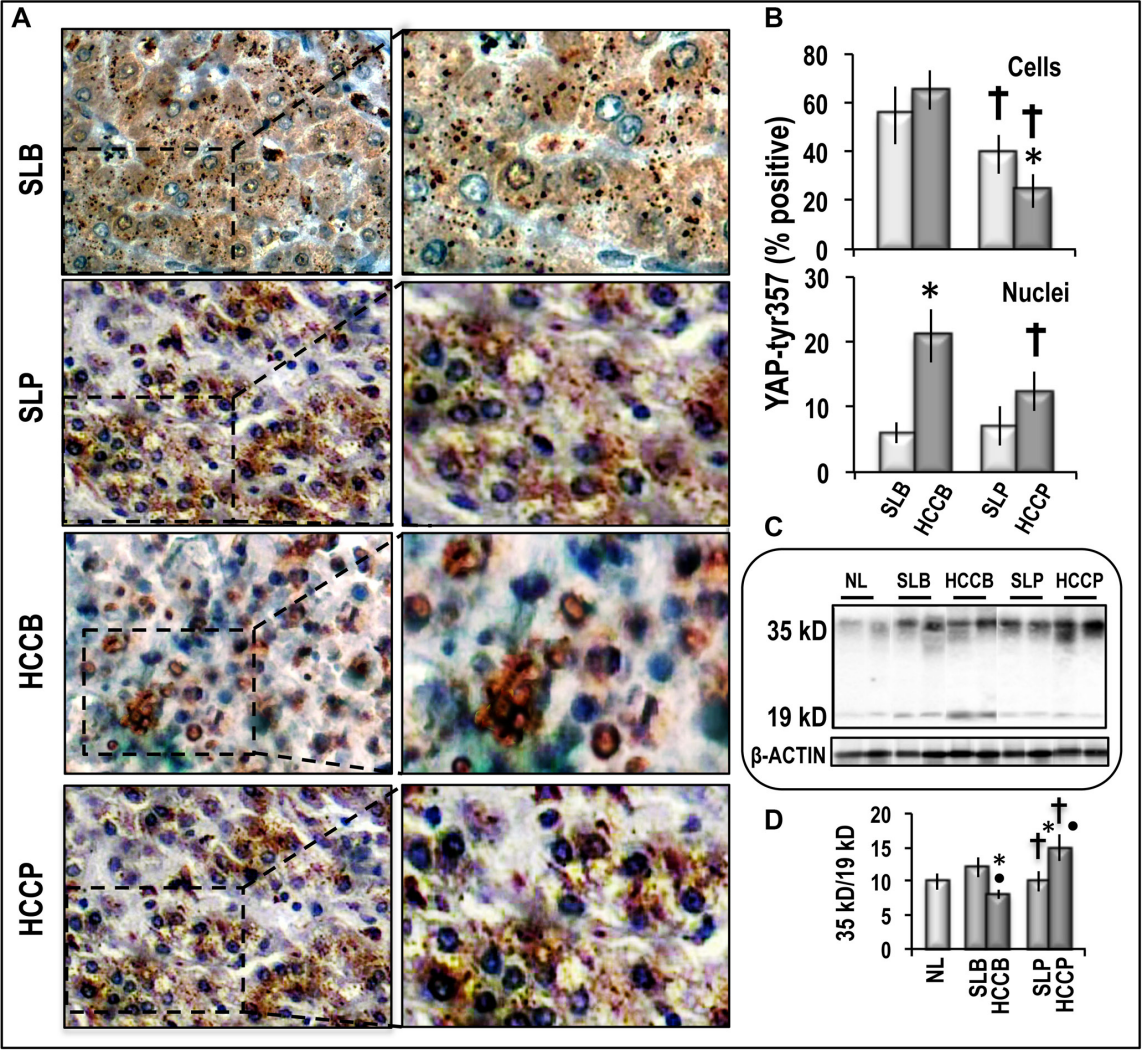
pYAP-tys357 expression and Caspase 3 cleavage in human HCC and corresponding surounding liver (**A**) Representative imaging of IHC staining of pYAP-tyr357 in HCCB and HCCP and SLs (100× and 400×). (**B**) Quantification of pYAP-tyr357 IHC staining: The IHC quantification was evaluated according to the percentage of cells with positive cytoplasms and/or nuclei (upper panel) or cells with positive nuclei (lower panel). Data are means (SD) of 3 different HCC and SL subtypes. Mann-Whitney test: Asterisk, HCC different from SL for at least *P* < 0.05. Dagger, HCCP/SLP different from HCCB/SL for *P* < 0.001. (**C**) Representative Western blots of Caspase 3 cleavage. (**D**) Chemiluminescence analysis: optical densities were normalized to β-actin levels and expressed in arbitrary units. Data are means (SD) of 5 experiments. Mann-Whitney test: Point, different from NL for *P* < 0.001. Asterisk, different from SL for *P* < 0.001. Dagger, HCCP/SLP different from HCCB/SLB for *P* < 0.001.

Figure 4 also shows that phosphorylated YAP1-tyr357 increased with respect to normal liver in surrounding liver and HCC, with highest values in HCCB. YAP1-tyr357 is a stable protein that enters the nucleus, displays high affinity to p73, and selectively co-activates, together with p73, proapoptotic genes [21]. Thus, the evaluation of the nuclear localization of phosphorylated YAP1-tyr357 in the different subtypes of HCC and correspondent SLs may give further insights on the effects of YAP1 post-translational deregulation in HCC. Figure 6A, 6B shows that the percentage of cells showing pYAP1-tyr357 immunoreactivity progressively decreased from HCCB to SLP and HCCP. HCCB also showed the highest nuclear localization of pYAP1-tyr357, whereas this localization was much lower in HCCP and was scanty in SLB and SLP. Accordingly, apoptosis (caspase 3 cleavage expressed as decrease in 36/19 kD ratio) was about 2-fold lower in HCCP than HCCB, whereas in SLs it did not significantly differ from that found in normal liver.

### YAP1 role in stem cell likeness of liver cancer

**Figure 7:**
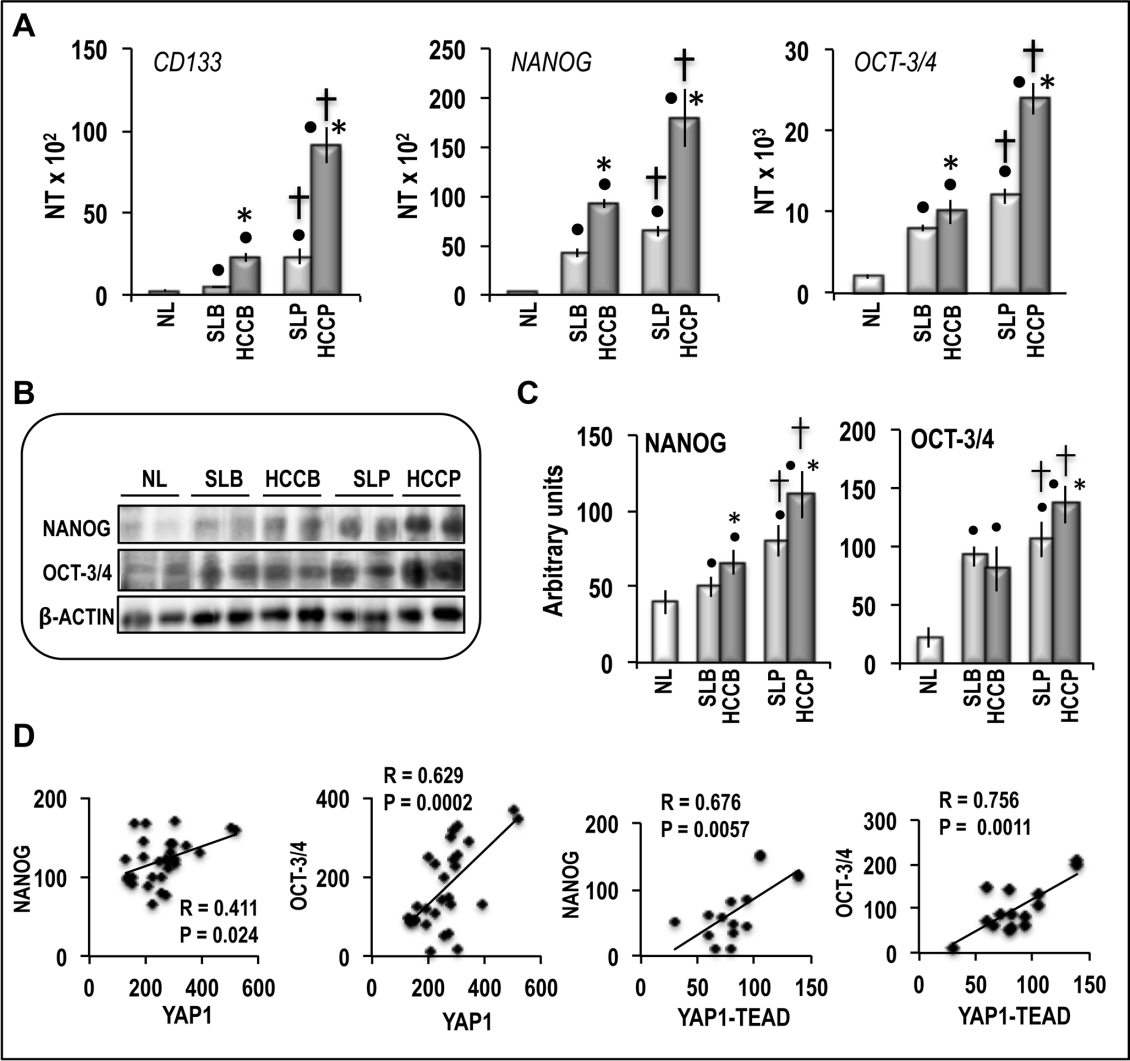
Expression of *CD133, NANOG and OCT3-3/4* in human NL, HCCB, HCCP, and corresponding SL (**A**) mRNA levels were determined by QRT-PCR. N Target (NT) = 2^-ΔCt^; ΔCt = Ct RNR18-Ct target gene. Data are means (SD) of NT of 5 NL and 20 of each HCC subtypes and SL. Mann-Whitney test: Point, different from NL for *P* < 0.001. Asterisk, different from SL for at least *P* < 0.01. Dagger, HCCP/SLP different from HCCB/SLB for *P* < 0.001. (**B**) Representative Western blots of NANOG and OCT-3/4. (**C**) Chemiluminescence analysis showing means (SD) of 5–15 experiments. Optical densities of the peaks were normalized to β-actin levels and expressed in arbitrary units. Mann-Whitney test: Point, different from NL for *P* < 0.001. Asterisk, different from SL for at least *P* < 0.05. Dagger, HCCP/SLP different from HCCB/SLB for at least *P* < 0.01. (**D**) Correlation of NANOG and OCT-3/4 levels with YAP1 and YAP1-TEAD complex in human HCCs. A total of 30 cases (15 HCCB and 15 HCCP) and a total of 15 cases (7 HCCB and 8 HCCP) were used for the Spearman's correlation analysis of YAP1 and YAP1-TEAD complex, respectively.

The aforementioned data envisage a connection of YAP1 post-translational regulation and YAP1-TEAD complex level with HCC aggressiveness. YAP-TEAD signaling was previously shown to activate the promoters of *OCT-3/4* and *NANOG* stem cell markers [22], and recent results [18] showed overexpression of CD90, a cancer stem cell marker, in HCC cell lines in which *YAP1* expression was stimulated as a consequence of *TAZ* inhibition. We thus examined the role of YAP1 in expression of stem cell markers in HCC prognostic subgroups. Figure 7A shows significant increases in stem cell markers *CD133,* *NANOG*, and *OCT-3/4* mRNA levels in HCCs and, at a lower extent, in the correspondent SLs, with respect to control, with highest values in HCCP. These results were partially confirmed by Western analysis, which showed progressive increase of NANOG and OCT-3/4 proteins from SL to HCC, and greatest values in HCCP (Figure 7B, 7C). Moreover, NANOG and OCT-3/4 protein levels were significantly correlated with YAP1 and YAP1-TEAD levels in HCC (Figure 7D).

**Figure 8:**
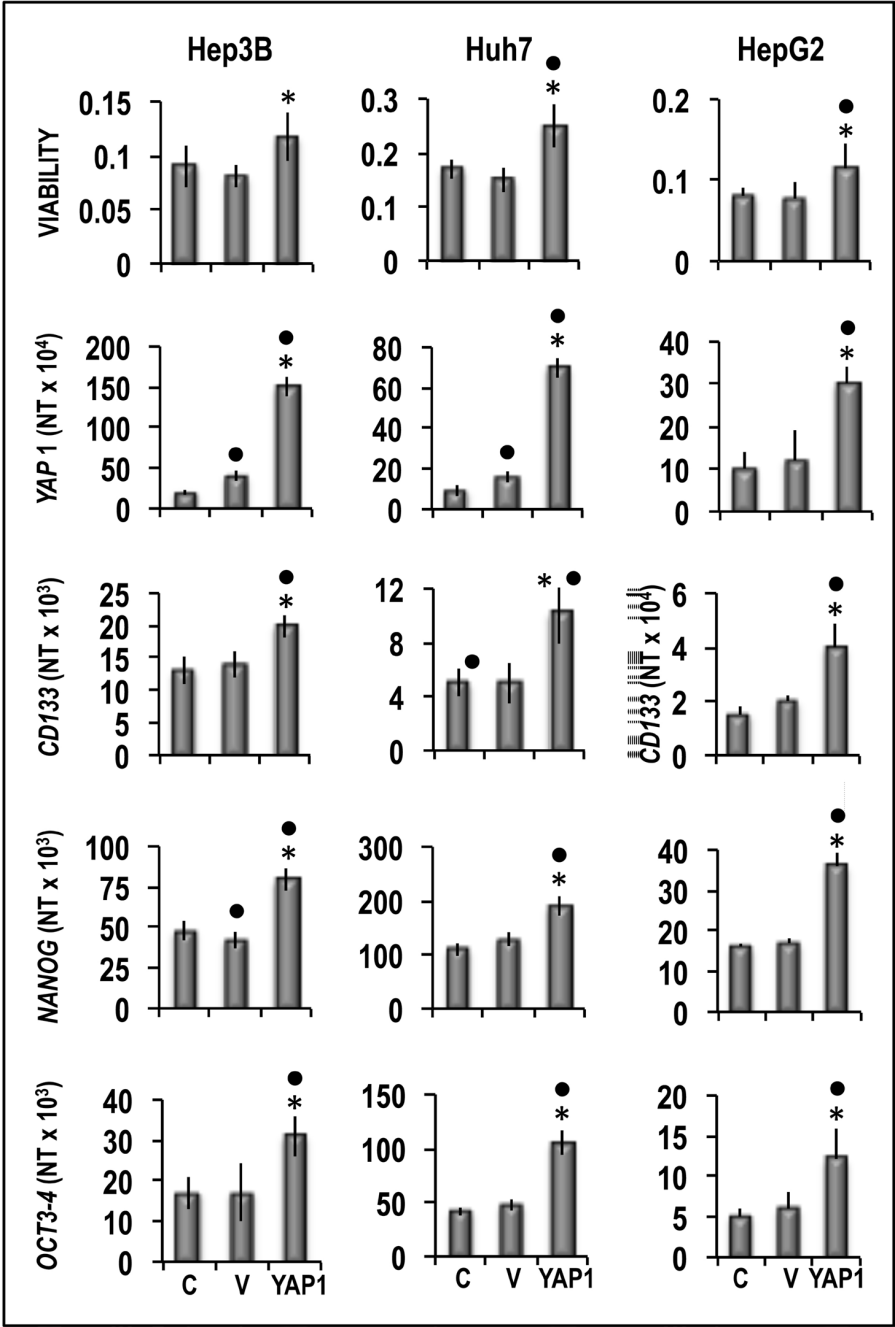
Effect of *YAP1* forced overexpression on the viability and expression of *CD133, NANOG* and *OCT3-3/4* of human Hep3B, Huh7, and HepG2 liver cancer cells The cells were transiently transfected with YAP1 cDNA in pCMV6 vector. Cell viability, and gene expression were determined in untransfected cells (C) or 48 h after transfection with empty vector (V) or 400 ng of *YAP1* cDNA. Data are means (SD) of three independent experiments of N-fold differences in mRNA expression relative to the RNR-18 expression, and named N Target (NT) = 2^-ΔCt^; ΔCt = Ct RNR18-Ct target gene. Mann-Whitney test. Viability: Point, different from C; asterisk, different from V for *P* < 0.001. Gene expression: Point different from C for at least *P* < 0.01; asterisk, different from V for *P* < 0.001.

**Figure 9:**
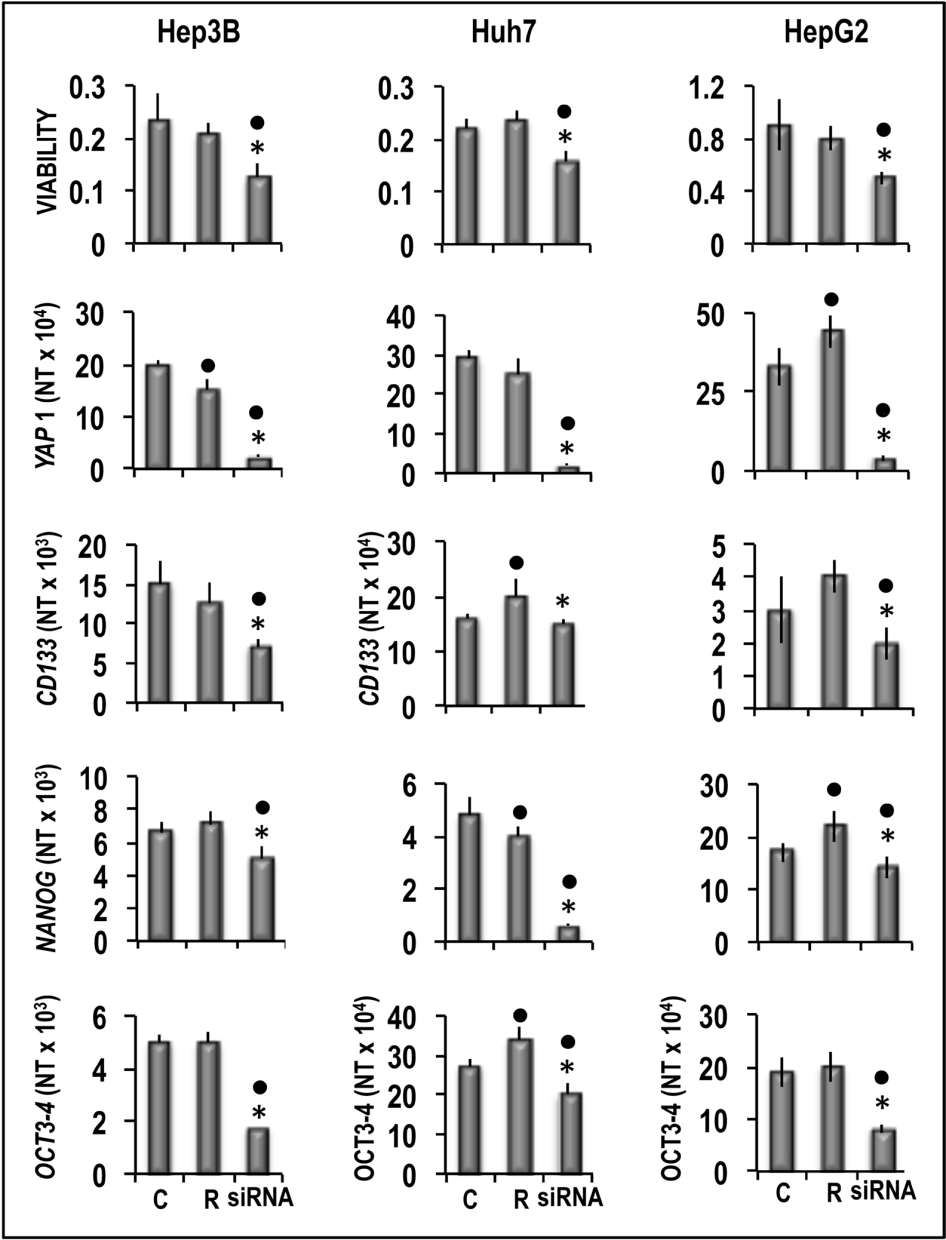
Analysis of the effect of *YAP1* inhibition by specific siRNA on the viability and expression of *CD133, NANOG* and *OCT3-3/4* of human Hep3B, Huh7, and HepG2 liver cancer cells The cells were transfected with RNAIRNT (R) or 50 nmol/L of YAP1 siRNA. Cell viability, and gene expression were determined in untransfected cells (C) or 48 h after transfection. Data are means (SD) of three independent experiments of N-fold differences in mRNA expression relative to the RNR-18 expression, and named N Target (NT) = 2^-ΔCt^; ΔCt = Ct RNR18-Ct target gene. Mann-Whitney test. Viability: Point, different from C; asterisk, different from R for *P* < 0.001. Gene expression: Point different from C for at least *P* < 0.01; asterisk, different from R for *P* < 0.001.

**Figure 10:**
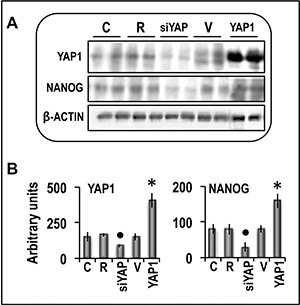
Analysis of the effect of YAP1 inhibition by specific siRNA or forced overexpression on the expression of NANOG protein in human Hep3B liver cancer cells The cells were transfected with RNAiMAX (R) or 50 nmol/L ng of YAP1 siRNA or with 400 ng of YAP1 cDNA in pCMV6 vector (V). Gene expression was determined in untransfected cells (C) or 48 h after transfection. (**A**) Representative Western blots. Protein lysates were immunoprecipitated with specific antibodies and separated by SDS-PAGE. (**B**) Chemiluminescence analysis showing means (SD) of 5 experiments. Optical densities of the peaks were normalized to β-actin levels and expressed in arbitrary units. Mann-Whitney test: Point, different from R; asterisk, different from V; *P* < 0.001.

**Figure 11:**
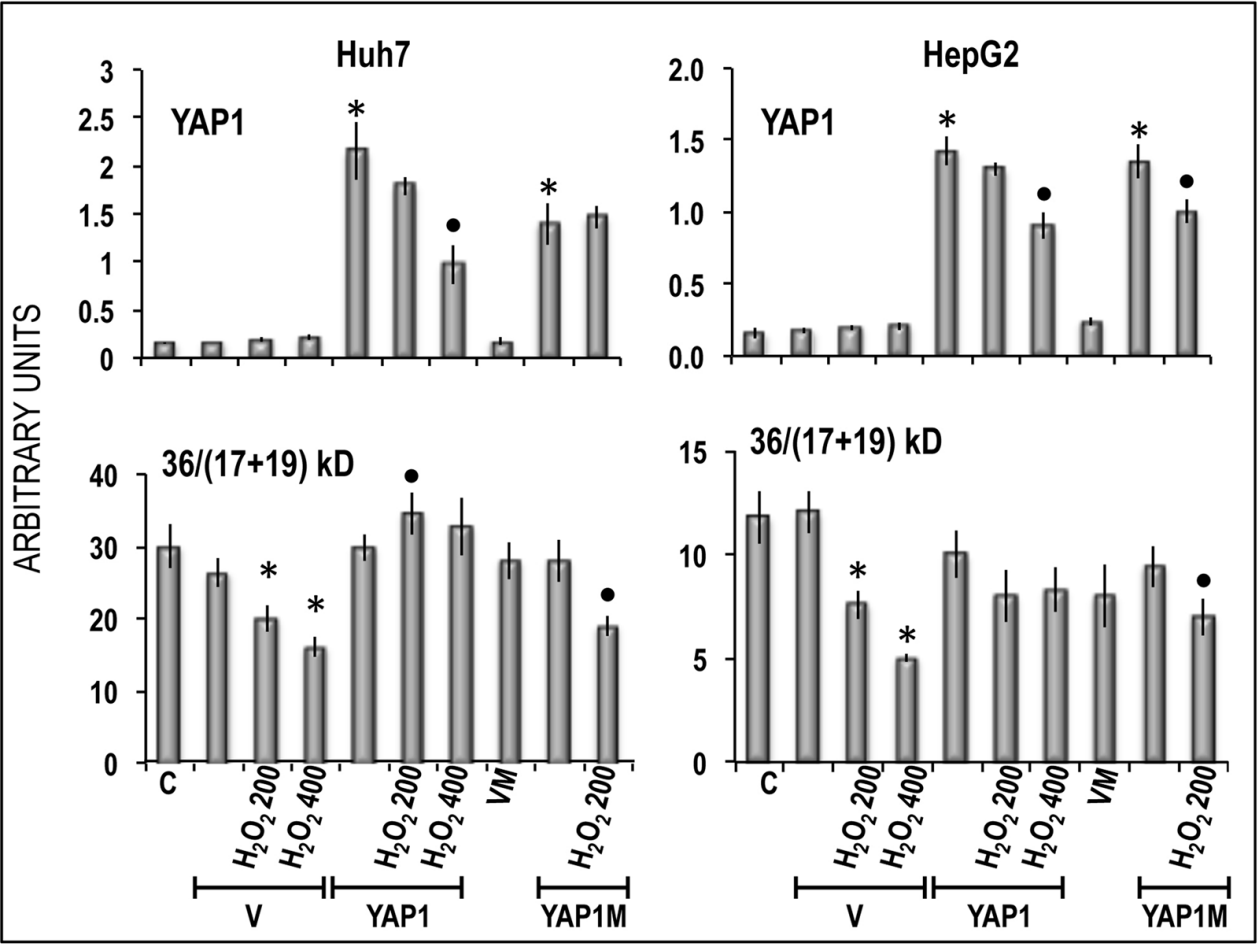
Inhibition by YAP1 of apoptosis induced in Huh7 and HepG2 cells by oxygen peroxide Apoptosis is expressed as decrease in the 36/(17+19) kD bands ratio indicating Caspase 3 cleavage. Chemiluminescence analysis: optical densities were normalized to β-actin levels and expressed in arbitrary units. Data are means (SD) of 3 experiments. Mann-Whitney test. Asterisks: difference from the appropriate vector for *P* < 0.001. Points: difference from YAP1/YAP1M for *P* < 0.001. Abbreviations: V, PCMV; VM, vector of mutated YAP1; YAP1M, mutated YAP1.

Functional experiments using Hep3B and Huh7 HCC cell lines and HepG2 hepatoblastoma cell line (Figure 8) showed that forced *YAP1* overexpression was associated with a significant increase in the viability and mRNA levels of the stem cell markers *CD133, NANOG,* and *OCT-3/4* in the three cell lines. In contrast, sharp decline of *YAP1* expression by a specific siRNA led to a decrease in cell viability associated with a significant restraint of *CD133,* *NANOG*, and *OCT-3/4* expression (Figure 9). These results were confirmed, at protein level, in Hep3B cells: NANOG underwent sharp decrease in cells treated with anti-YAP1 siRNA and significant increase consequent to forced YAP1 overexpression (Figure 10A, 10B). Furthermore, YAP1 transfection protected significantly Huh7 and HepG2 cell lines from apoptosis induced by 200 and 400 μM H_2_O_2_, although the higher H_2_O_2_ concentration inhibited by 50–60% YAP1 expression (Figures 1 and 2). Very low/no protection from apoptosis, induced by 200 μM H_2_O_2_, occurred in the cells transfected with mutant YAP1 (Supplementary Figure 1 and Figure 11).

The modulation of YAP1 levels strongly influenced *in vitro* migration and invasivity of Huh7 cells. Indeed, YAP1 overexpression resulted in enhanced cell migration *in vitro* that, differently from the cells transfected with the empty vector, led to almost complete reconstitution of the cell monolayer 36 h after the wound was generated in confluent cells. In contrast, the monolayer was not completely reconstituted even 48 h after wounding in cells treated with YAP1 siRNA (Supplementary Figure 2). Cell invasivity test revealed that forced YAP1 overexpression was associated with a 2-fold increase in cell invasion through polycarbonate membrane, whereas inhibition of YAP1 expression led to an about 5-fold decrease in cell invasion (Supplementary Figure 3).

**Figure 12:**
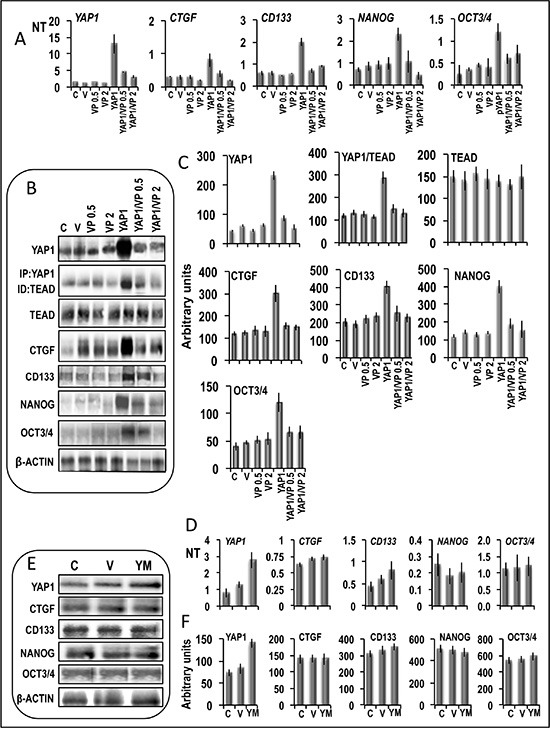
Evidence of the involvement of the YAP1-TEAD complex in the regulation of the expression of the stem cell markers (**A–C**) Effect of 0.5 and 2 μM verteporfin on expression of stem cell markers in Huh7 cells transfected with *YAP1*. (A) mRNA levels were determined by QRT-PCR. N Target (NT) = 2^-ΔCt^; ΔCt = Ct RNR18-Ct target gene. Data are means (SD) of 3 experiments. (B) Representative Western blots. (C) Chemiluminescence analysis showing means (SD) of 4 experiments. Optical densities of the peaks were normalized to β-actin levels and expressed in arbitrary units. Mann-Whitney test: YAP1 vs. V, *P* < 0.001, and YAP1/VP0.5/2 vs YAP1, *P* < 0.001 for all genes tested, for both quantitative RT-PCR and chemiluminescence analysis. (**D–F**) Expression of stem cell markers in Huh7 cells transfected with mutant YAP-S94A/S127A (YM). (D) mRNA levels were determined by QRT-PCR. (E) Representative Western blot and (F) cheliluminescence analysis showing means of 3 experiments. Optical densities of the peaks were normalized to β-actin levels and expressed in arbitrary units. Mann-Whitney test: YM vs. V, *P* < 0.001 for YAP1; not significant for all other genes. Abbreviations: C, control; V, empty vector; VP0.5, VP2, verteporfin 0.5 and 2 μM. YM, mutated YAP1. Vertperfin was dissolved in DMSO. Results with DMSO alone did not differ from C and were not included in the figure.

Previously, it has been shown that TEAD mediates YAP-effect on gene induction and growth [23]. In order to demonstrate the involvement of YAP1-TEAD complex in the activation of stem cell markers in liver cancer cells, we evaluated the effect of verteporfin, which was reported to impede the YAP1-TEAD complex formation [24] and does not inhibit, at low doses, basal YAP expression [25]. In accordance with previous data, we found that the addition of 0.5 and 2 μM verteporfin to HepG2 cells (Figure 12) did not significantly alter the basal mRNA and protein levels of all genes tested. However, verteporfin inhibited the increase in *YAP1* mRNA and protein levels and YAP1-TEAD complex level in *YAP1* transfected cells, without modifying TEAD levels, and was associated with relatively low/no increases in *CTGF, NANOG, OCT-3/4,* and *CD133* mRNA and protein levels (Figure 12A–12C). Although these observations confirm a link between YAP1 upregulation and stem cell marker overexpression, they do not exclude the toxicity of even low verteporfin doses on YAP1 expression, which could hamper to evaluate the actual role of the decrease in YAP-TEAD complex. To rule out this possibility, HepG2 cells were transfected with mutated YAP1 (YAP-S94A/S127A; Figure 12D–12F), which does not form complex with TEAD [23]. No significant increases in mRNA and proteins levels of *CTGF* and stem cell markers occurred in these conditions.

### YAP1 overexpression induces the expression of genes putatively involved in cancer stemness

**Figure 13:**
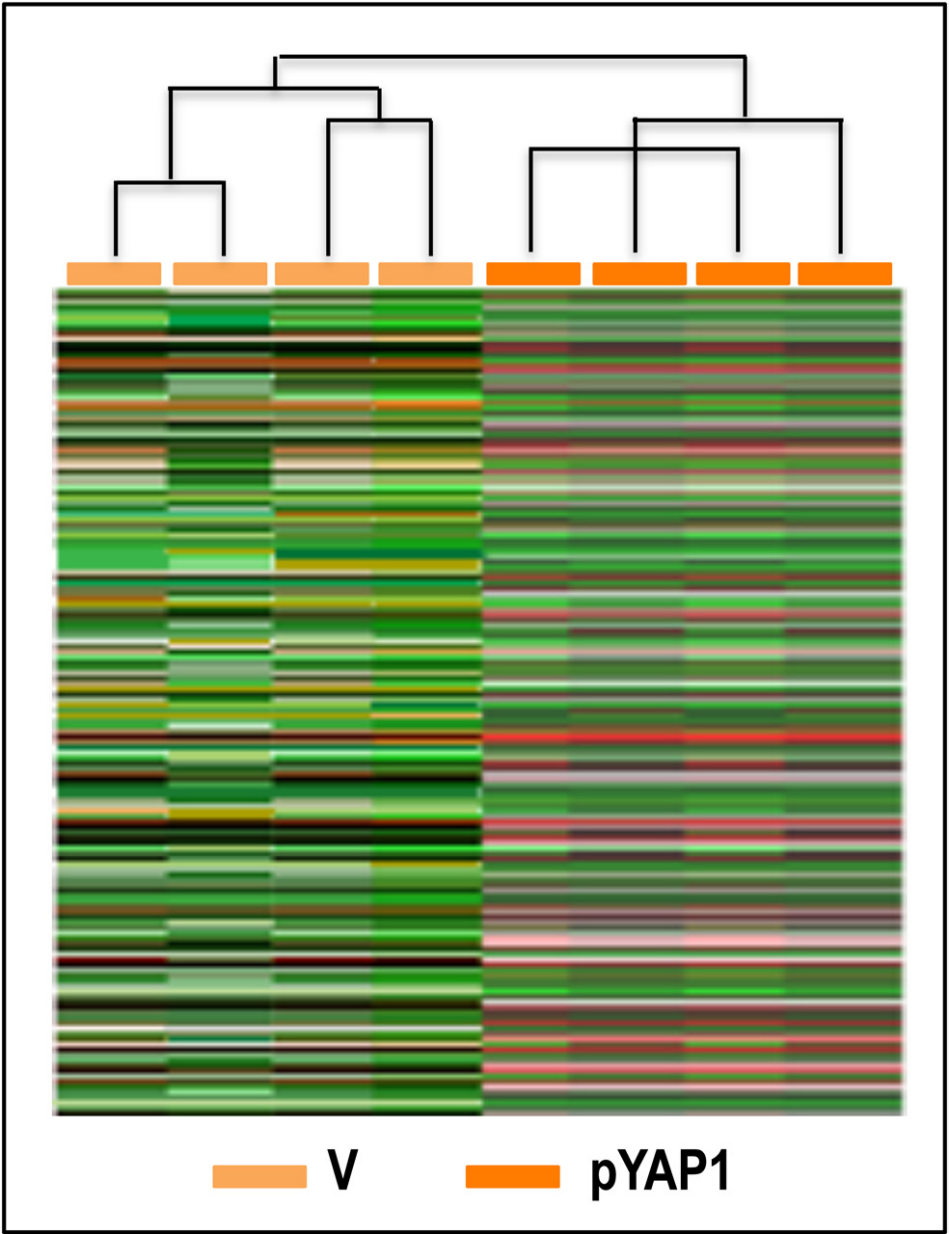
Unsupervised hierarchical cluster analysis of gene expression patterns of Huh7 cells Microarray experiments with RNA from 4 independent Huh7 cell cultures, transfected with YAP1 cDNA or empty vector (V) were made. 136 gene features, showing more than 1.5-fold difference compared to median expression value in 4 arrays, were selected for cluster analysis. Expression values were Log 2 transformed before clustering. Rows represent individual genes and columns represent each tissue.

**Figure 14:**
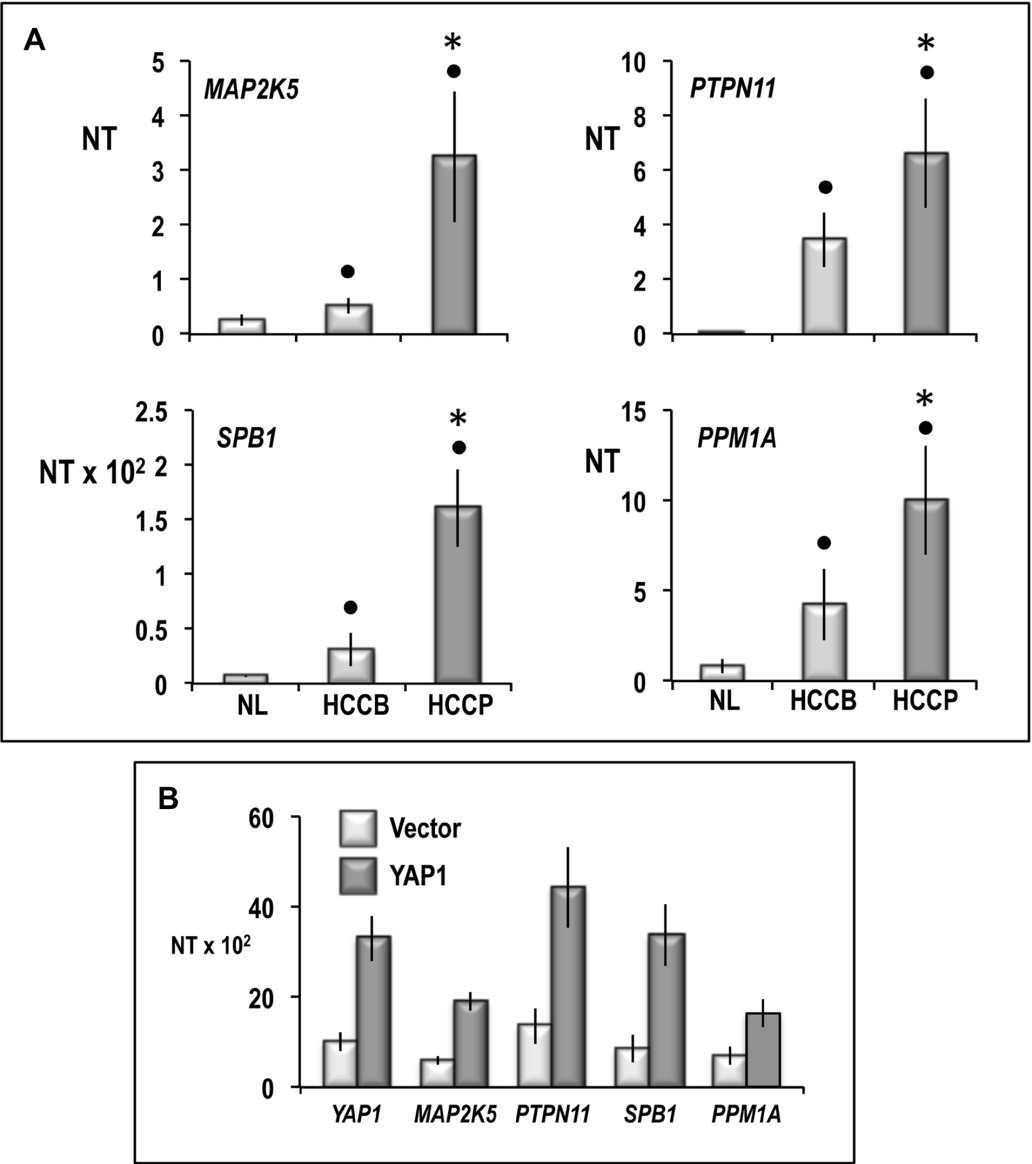
Expression of *MAP2K5, PTPN11, SPB1*, and *PPM1A* in human normal liver (NL) and HCCB and HCCP (A) and HepG2 cells transfected with YAP1 (B) mRNA levels were determined by QRT-PCR. N Target (NT) = 2-ΔCt; ΔCt = Ct RNR18-Ct target gene. Data are means (SD) of NT of 5 NL and 10 of each HCC subtypes and corresponding SL and of 3 independent experiments with HepG2 cells. Mann-Whitney test: solid tumors: Point, different from NL for *P* < 0.001. Asterisk, different from HCCB for at least *P* < 0.01. HepG2 cells: YAP1 vs vector, *P* < 0.001 for all genes tested.

To further determine the cellular signaling network activated by YAP1 in HCC, gene expression profile analysis was carried out in four independent YAP1-transfected Huh7 cell cultures and four cultures transfected with empty vector. Cluster analysis of 136 gene features showing more than 1.5-fold difference compared to median expression value in 4 arrays, revealed two distinctive gene expression patterns, the first of which included Huh7 cells transfected with empty vector, and the other YAP1 transfected cells (Figure 13). Data analysis using high statistical stringency revealed that the expression of 43 genes significantly differed between transfected cells and their controls (Table 2). Genes activated, in YAP1-transfected cells, were involved in signal transduction and cell proliferation (*CTGF*, *NARG2, MAP2K5, SPSB1, SFSR10, PDE2A, PTPN11, PTPRM, PEX14, CAPRIN 1, CEP5S, CSPP1, LGALS7, IL1A*), and cell metabolism (*LDHA, AADACL1*). Genes inhibited (Table 2) included genes involved in signal transduction (*CFM2, CDC42SE2, HHIP*) and metabolism (*ALG3, PDHA2*). To test the behavior of genes whose expression was influenced by changes in *YAP1* expression *in vivo*, the levels of *MAP2K5, PTPN11, SPB1*, and *PPM1A* mRNA were determined in HCC prognostic subgroups. A significant increase in expression of these four genes occurred in HCC with respect to normal liver, with highest values in HCCP (Figure 14A). The occurrence of a link between *YAP1* and *MAP2K5, PTPN11, SPB1*, and *PPM1A* expression was confirmed by the observation that forced *YAP1 e*xpression in HepG2 cells led to a 3–3.7 fold rise in their mRNA levels (Figure 14B).

**Table 2:** Deregulated genes in Hep3B cells upon YAP forced overexpression identified by microarray analysis^1^

Gene symbol	Description	Log-ratio	Parametric *P* value
**Up-regulated genes**			
*Signal transduction*			
CTGF*	connective tissue growth factor	0.7628	0.0064
NARG2*	N-methyl-D-aspartate receptor regulated 2	0.5586	0.0183
MAP2K5* §	mitogen-activated protein kinase kinase 5	11′199	0.0000
SPSB1* §	SPRY domain-containing SOCS box protein 1	0.6239	0.0025
SFSR10*	splicing factor, arginine/serine-rich 10 (transformer 2 homolog, Drosophila)	0.4996	0.0021
PPM1A* §	protein phosphatase 1A, magnesium-dependent	0.3134	0.0357
PTPN11* §	protein tyrosine phosphatase, non-receptor type 11	0.3143	0.0155
PTPRM §	protein tyrosine phosphatase, receptor type, M	0.7708	0.0057
PEX14*	peroxisomal biogenesis factor 14	0.5018	0.0292
PDE2A* §	phosphodiesterase 2A, cGMP-stimulated	19′609	0.0000
*Cell proliferation*			
CAPRIN1*	cell cycle associated protein 1	0.3910	0.0011
CEP55	centrosomal protein 55 kDa	0.6724	0.0040
CSPP1*	centrosome and spindle pole associated protein 1	0.3407	0.0127
LGALS7	lectin, galactoside-binding, soluble, 7 (galectin 7)	0.4825	0.0412
IL1A	interleukin 1, alpha	0.4008	0.0018
CSRP1	cysteine and glycine-rich protein 1	0.3151	0.0211
IRF2 §	interferon regulatory factor 2	0.6768	0.0342
*Metabolism*			
LDHA §	lactate dehydrogenase A	0.2179	0.0492
AADACL1*	arylacetamide deacetylase-like 1	0.3646	0.0006
HGSNAT*	heparan-alpha-glucosaminide N-acetyltransferase	0.5291	0.0253
MCCC1	methylcrotonoyl-Coenzyme A carboxylase 1 (alpha)	0.4430	0.0013
*Varia*			
RBM35B*	RNA binding motif protein 35B	0.4840	0.0029
RTTN	rotatin	0.3821	0.0183
SH3KBP1 §	SH3-domain kinase binding protein 1	0.4996	0.0021
CTDSPL2*	carboxy-terminal domain small phosphatase like 2	0.5825	0.0079
NUP155	nucleoporin 155 kDa	0.4724	0.0447
Down-regulated genes			
*Signal transduction*			
CCM2*	cerebral cavernous malformation 2	−0.5444	0.0018
CDC42SE2	CDC42 small effector 2	−0.7453	0.0019
ACTR5	ARP5 actin-related protein 5 homolog (yeast)	−0.4928	0.0012
HHIP	hedgehog interacting protein negative regulation of	−0.4907	0.0014
MYBL1	v-myb myeloblastosis viral oncogene homolog (avian)-like 1	−0.6037	0.0160
TLE3*	transducin-like enhancer of split 3 (E(sp1) homolog, Drosophila)	−0.4688	0.0406
RAB4A	RAD9 homolog B (S. cerevisiae) protein transport, small GTPase mediated signal transduction	−0.3416	0.0095
*Metabolism*			
ALG3*	asparagine-linked glycosylation 3 homolog (S. cerevisiae, alpha-1,3-mannosyltransferase)	−0.3714	0.0188
PDHA2	pyruvate dehydrogenase (lipoamide) alpha 2	−0.4227	0.0101
PRSS7	protease, serine, 7 (enterokinase)	−0.6448	0.0000
*Varia*			
DERL3	Der1-like domain family, Derlin-3	−0.3761	0.0088
ECE2*	endothelin converting enzyme 2	−0.4110	0.0009
ITGA2	integrin alpha 2	−0.3717	0.0314
ORM2*	Orosomucoid 2	−0.3725	0.0323
TERT	telomerse reverse transciptase (telomere maintenance)	−0.3387	0.0307
YIPF3*	Yip1 domain family, member 3	−0.4741	0.0002
PSME2*	proteasome activator subunit 2	−0.3550	0.0144

1 Asterisk: presence of the TEAD binding sites in gene promoters. Paragraph: genes involved in stem cell activation.

## DISCUSSION

YAP1 overexpression was suggested to be an independent prognostic marker for HCC [4, 26]. In line with these findings, our results show that the upregulation of the *AREG/EGFR/YAP1/CTGF* pathway is linked to HCC prognosis. Further, our data clearly indicate, for the first time, that the effects of YAP1 overexpression on HCC prognosis are largely influenced by differences in YAP1 post-translational deregulation. Indeed, higher YAP1 phosphorylation at ser127 and its ubiquitination, probably preceded by further phosphorylation of YAP1-ser127 [4, 5, 27], occurred in HCCB, as compared to HCCP. Moreover, HCCB mainly exhibited YAP1 phosphorylation at ser357, which is associated with its nuclear translocation followed by p73 activation, and apoptosis [4, 5, 22, 26]. This could contribute to the better prognosis of this HCC subgroup. In contrast, lower phosphorylation at ser127 was prevalently associated with the formation of the cytoplasmic YAP1-14-3-3 complex, while the presence of elevated levels of YAP1-TEAD complex indicated a consistent nuclear translocation of unphosphorylated YAP1, in HCCP. The YAP1-TEAD complex activates numerous growth-related genes in HCC [27]. Accordingly, we found a significant positive correlation of YAP1 and YAP1-TEAD levels with the expression of the proliferation marker Ki67, and the progression markers CTGF [28, 29] and MDK [19, 20], in HCC.

Our results confirm previous observation that YAP1 over-expression is an early event in rat liver carcinogenesis [9]. They also show higher YAP1 expression in preneoplastic and neoplastic lesions of F344 rats, genetically susceptible to hepatocarcinogenesis, compared to resistant BN rats. Previous work in our laboratory [2] demonstrated that, similarly to the development of human HCC [30], the development of rat preneoplastic and neoplastic liver is under control of low-penetrance cancer modifier genes. Present results indicate that YAP1 post-translational deregulation favoring its ubiquitination, activation of p73, and apoptosis, is significantly higher in well-differentiated and slow-growing HCC of the genetically resistant BN strain, whereas YAP1 post-translational regulation seems to favor HCC fast growth in susceptible F344 rats. This suggests that YAP1 post-translational deregulation is under control of HCC modifier genes, which could contribute to determine the relative amounts of YAP1 triggering HCC growth and progression, and the amounts designed to be ubiquitinylated or to activate apoptogenic mechanisms. The similarity of the YAP1 post-translational deregulation in human and rat HCC, and of the genetic model regulating hepatocarcinogenesis in human and rat [2], suggests that YAP1 post-translational deregulation is under an analogous genetic control mechanism in both species.

Recent studies showed that Hippo/Yap signaling controls the epithelial progenitor cell proliferation and differentiation [31]. Our observations clearly show a link between the overexpression of *YAP1* and the acquisition of some stem cell properties by HCC. Specifically: (a) both *YAP1* and *CD133, NANOG* and *OCT-3/4* stem cell markers are overexpressed in HCC, and their expression is higher in more aggressive tumors exhibiting higher growth rate and lower apoptosis; (b) NANOG and OCT-3/4 protein levels are statistically correlated with YAP1 and YAP1-TEAD levels in HCC; (c) forced *YAP1* overexpression in liver tumor cell lines induces consistent increase of *CD133, NANOG* and *OCT-3/4* expression, cell viability, migration and invasivity, whereas *YAP1* inhibition by specific siRNA leads to opposite changes; (d) stem cell markers overexpression does not occur in cancer cell lines transfected with mutant YAP1 that does not form complexes with TEAD [23]. Remarkably, microarray analysis showed that forced *YAP1* overexpression in Huh7 cells resulted in increased expression of genes that mediate the acquisition of a stem cells phenotype in cancer of liver and/or other tissues, including *MAP2K5* [32, 33]*, PPM1A* [34], *PTPN11* [35], *PTPRM* [36], *PDE2A* [37] *IRF2* [38], *LDHA* [39] *SH3KBP1* [40] and *SPSB1* [41]. Interestingly, the expression of *MAP2K5, PPM1A, PTPN11,* and *SPSB1* revealed a sharp increase especially in more aggressive HCCP exhibiting highest expression of stem cell markers, as well as in HepG2 cells transfected with YAP1. Taken together, these findings strongly suggest that the YAP1/TEAD complex may induce, both directly and indirectly, several changes leading to the acquisition of some stemness features by HCC cells.

Microarray analysis also revealed that forced *YAP1* overexpression transcriptionally activates a variety of genes involved in signal transduction and cell proliferation. Among them, *PPM1A* encodes a phosphatase that dephosphorylates nuclear exporter RanBP3 at ser58, thus stimulating nuclear exclusion of SMAD2/3 [42]. *SFRS10* [43], *CAPRIN1* [44], and *CEP55* [45] are implicated in ovarian, breast and bladder carcinogenesis, respectively. *CSPP1* is a mitosis regulator [46]; *LGALS7* [47] and *PDE2A* [48] are overexpressed in different cancer types; *LDHA* encodes one of the major glycolysis enzymes. Of note, glycolytic ATP production in HCC is correlated with tumor cell aggressiveness [1].

Among inhibited genes, *CDC42SE2* controls the activity of cell division cycle protein (CDC42), which exhibits oncosuppressor activity [49]. *HHIP* encodes an inhibitor of the Hedgehog pathway, implicated in HCC pathogenesis [1, 50]. *PDHA2* encodes pyruvate dehydrogenase. The overexpression of the E1α subunit of pyruvate dehydrogenase complex is apoptogenic for HCC cells [51]. *DERL3* loss leads to *SLC2A1* (glucose transporter 1, GLUT1) overexpression, which contributes to the Warburg phenomenon of tumor cells [52]. *ITGA2* inhibition is associated with non-invasive prostatic cancer [53] and reduced cell migration in colorectal cancer [54]. The TEAD consensus sequence is present in the promoter of several genes whose expression was increased or decreased in YAP1-transfected cells (Table 2). Surprisingly, the *TERT* gene, generally overexpressed in HCC cells [55], was inhibited in YAP1-transfected cells. It must be considered, in this respect, that individual functional elements in transcriptional activation domains are responsible for activating specific cellular genes by different transcription factors, in a context-specific manner [56, 57]. Therefore, it may be hypothesized that signaling pathways different from Hippo/YAP signaling may prevalently regulate some genes, in the absence of forced YAP1 overexpression. Further work is necessary to clarify this apparent discrepancy. Overall, gene expression profiles of Huh7 cell transfected with *YAP1* strongly suggest that this gene may influence HCC cell growth and progression by complex mechanisms involving numerous genes/signaling pathways regulating the proliferation, stemness and aggressiveness of HCC.

In conclusion, our data indicate that YAP1 overexpression and post-translational deregulation are genetically controlled and contribute to determine a phenotype susceptible to HCC [2] and HCC prognosis. Our results support a link between *YAP1* overexpression and acquisition of a stem cell trait by HCC and the hypothesis [58] that *YAP1* upregulation in HCC exhibits a critical oncogenic role in a complex signaling network triggering fast growth and invasiveness.

## MATERIALS AND METHODS

### Animals and treatments

F344 and BN rats (Charles-River-Italia, Calco, Italy) were fed, housed, and treated according to the “resistant hepatocyte” protocol [59], consisting of a 150-mg/kg intraperitoneal dose of diethylnitrosamine followed by 15 days of feeding a 0.02% 2-acetylaminofluorene-containing hyperprotein diet, with a partial hepatectomy at the midpoint of this feeding regime. Preneoplastic liver (6 weeks after initiation), early dysplastic nodules (15 weeks) and late dysplastic nodules (32 weeks), and HCCs (57–60 weeks) were used. Animals received human care, and study protocols were in compliance with the National Institutes of Health guidelines for use of laboratory animals. Rats were killed by bleeding through thoracic aorta, under metedomidine anesthesia. Freshly removed livers were serially sectioned with ~0.5 cm intervals. Fifteen and 32 weeks after initiation, dysplastic nodules macroscopically identified by their sharp grayish-white color, were scooped out from the liver, free of surrounding parenchyma (as verified by histological control). HCCs were collected from F344 and BN rats leaving out a small rim of neoplastic tissue. Only DNs with diameter ≧ 0.03 cm^3^ were collected from both rat strains and split in half. One half of this material was processed for histology, histochemistry, and immunohistochemistry, and the other half was stored at −80°C. Histological (HE staining), histochemical (silver staining of reticulin) and immunohistochemical (glutamine synthase immunostaining) criteria were used, in addition to morphology, to classify liver lesions according to the published criteria [60–62], (data not shown).

### Human tissue samples

Six normal livers, 20 HCCP and 20, HCCB and corresponding surrounding non-tumor livers were used. Patients' clinicopathological features are shown in Table 1. Liver tissues were archival samples kindly provided by the Department of Surgery “Pietro Valdoni”, University of Rome “La Sapienza”, and the Department of Surgery, University of Sassari. Informed patients' consent and Institutional Review Board approval was obtained at these Departments.

### Cell lines and treatments

Certified HepG2 human hepatoblastoma, Hep3B and HuH7 human HCC cell lines were obtained from ATCC and maintained as monolayer cultures in Dulbecco's modified Eagle medium containing 10% FBS, at 37°C. A total of 0.8 × 10^6^ cells were seeded in 6 cm dishes and transfected with small interfering RNA (siRNA) duplexes specific to human YAP1. siRNA and scramble oligonucleotide (final concentration 50 nmol/L) were transfected using the RNAiMax, Invitrogene kit (Life technology, CA, USA). For transient transfection experiments, HepG2, Hep3B, and Huh7 cell lines were seeded and incubated 24 h before transfecting with pCMV6_YAP1 (400 ng of YAP1 cDNA), mutant YAPS127AS94A [23] (a kind gift Dr. Xin Chen, University of California, San Francisco), or pCAMV6-empty Vectors (Origene, Rockville, MD, USA) by lipofectamine 2000 Reagent (Life technologies Corp.) according to manufacturer's protocol. When indicated, verteporfin (0.5 or 2 μM in DMSO) or DMSO were added to the reaction mixture used for YAP1 transfection. For induction of apoptosis, H_2_O_2_ was added to cell cultures, at 200 and 400 μM final concentrations, 6 hour after transfection with YAP1. After 24 h incubation, Caspase 3 cleavage was determined by immunoblotting as a measure of apoptosis and expressed as decrease in the ratio 36/(19 + 17) kD bands.

### Proliferation and viability indices

Proliferation and progression indices were evaluated in human HCC by determining Ki-67 and MDK expression, respectively. Cell viability of liver tumor cell lines was determined by MTT test (Sigma, St. Louis, USA). For wound-healing assay with YAP1-transfected cells, Huh7 cells were seeded into 6-well plates and cultured to confluence. Cells monolayers were then wounded with sterile pipette tips and washed with PBS. Pictures were acquired at the times indicated using a fluorescence microscope. Cell invasivity was analyzed by the Cytoselect 24-well cell invasion kit (Cell Biolabs, San Diego, USA), with 50.000 Huh7 cells/well.

### Quantitative real-time RT-PCR

Real-Time was performed on cDNA obtained accordingly to High Capacity c-DNA Reverse Transcription Kit (Applied BioSystem, CA, USA). PCR reactions were performed with 75–300 ng of cDNA, using an ABI Prism 7500 and Quantitect SYBR Green PCR kit & Quantitexct Primer Assay (Qiagen Gmbh, Hilden, Germany), as published [63].

### Western blot analysis

Hepatic tissue samples and cell suspensions from cultured cancer cells were homogenized in lysis buffer [30 mM Tris (pH 7.5), 150 mM NaCl, 1% NP-40, 0.5% Na deoxycholate, 0.1% SDS, 10% glycerol, and 2 mM EDTA] containing the Complete Protease Inhibitor Cocktail (Roche Molecular Biochemicals, Indianapolis, IN) and sonicated. Protein concentrations were determined with the Lowry-Folin assay (Sigma, St. Louis, USA) using bovine serum albumin as standard. Proteins were cleaned by binding G-sepharose beads & IgG normal control (rabbit, goat & mouse). For determination of YAP1-14-3-3 and YAP1-TEAD complexes, proteins were immunoprecipitated by YAP-goat antibody and western blot was performed using 14-3–3 or TEAD1 primary rabbit antibodies. Membranes were probed with the antibodies shown in Table 3 and processed as reported [61]. Each primary antibody was followed by incubation with horseradish peroxidase-secondary antibody diluted 1:5000 for 1 h and then revealed with the Super Signal West Pico Chemiluminescence Substrate Kit (Pierce Chemical Co., New York, NY). For each protein, densities were calculated by ImageQuaNT 5.1 software (GE Healthcare, Piscataway, NJ), normalized to β-Actin (Santa Cruz Biotechnology, Santa Cruz, CA; dilution 1:10000) levels, and mean values evaluated for statistical significance.

**Table 3:** Antibodies used for immunoprecipitation and Wester analyses

Antibody	Species (Antibody Titres)	Company	Immunogen
OCT-3/4 (A-9)	Mouse (1:50)	Santa Cruz; 365509	a.a 16-45 near the N-terminus
NANOG (1E6C4)	Mouse (1:100)	Santa Cruz; 293121	a.a 20-166
PAN-TEAD	Rabbit (1:1000)	Cell Signaling; 8526	D3F7L
CD133/1	Mouse (1:150)	Macs; W6B3C1	Clone W6B3C1
YAP	Mouse (1:400)	Abnova; H00010413-M01	53 a.a~161 a.a
YAP (C-20)	Goat	Santa Cruz; 17141	C-20 peptide Near the C-terminus of Yap
p-YAP S127	Rabbit (1:1000)	Cell Signaling; 4911	Ser 127
p-YAP Y357	Rabbit (1ug/ml)	Abcam; AB62751	Tyr357 in C-terminal amino acids 351-362 of Human YAP1
CASPASI 3	Rabbit (1:1000)	Cell Signaling; 9662	KLH peptide corresponding to residues surrounding the cleavage site of caspase3
p-p73 y99	Rabbit (1:1000)	Abcam; AB38457	Tyr 99
14-3-3 σ	Mouse (1:300)	Santa Cruz; 166473	E-11 epitope mapping between a.a 8-38 at the N-terminus of 14-3-3
Ub (P4D1)	Mouse (1:500)	Santa Cruz; 8017	a.a 1-76 representing full lenght Ub
CTGF	Rabbit (1:500)	Abcam; 5097	Residues 150-250

### Microarray analysis

High-quality RNA from 4 independent Huh7 cell cultures transfected with 400 ng of YAP1 cDNA and 4 control cell lines transfected with vector alone, were used to obtain fluorescently labeled complementary double-stranded DNA (cDNA). cDNA was labeled with Cy3 or Cy5, hybridized using Agilent *In situ* Hybridization Kit-plus (Agilent Technologies, Wilmington, DE). Microarray experiments were made with Agilent Human G4131F 4 × 44 K. Fluorescence intensities were measured using ImaGene 8.0 software and analyzed by Nexus Expression software (Biodiscovery, El Segundo, CA). To select the spot subsets, we followed the criterion of minimum variance in quadruplicate fluorescence ratio measurements, when the fluorescence signal was higher than 0.3% of the measurable total signal dynamics range above background in both channels of the hybridization. For normalization, the intensity of each spot was divided by average intensity of housekeeping genes. Genes showing more than 1.5- fold difference compared to median expression value in more than 4 arrays, were selected for cluster analysis. The algorithm based on Pearson correlation coefficients was used for hierarchical cluster analysis. K-mean clustering analysis, and visualization of analyzed data were performed as described [64].

### Statistical analysis

Data are expressed as means ± SD. GraphPad Prism 5.01 (www.graphpad.com) was used to evaluate, by Tukey-Kramer test, the significance of differences between means of QPCR and Western blot analyses of rat samples, and calculate the correlation coefficient (R) by multiple regression analysis. The expression of human HCC subgroups were evaluated by the Mann-Whitney *U*-test. Statistical analysis of microarray results was performed by parametric Student's *t*-test and the False Discovery Rate (FDR) method to correct *P*-values and control false identifications, using the BRB Array Tools (http://linus.nci.nih.gov/BRB-Array.Tools.html). Values of *P* < 0.05 were considered significant.

## SUPPLEMENTARY MATERIALS FIGURES


