# Supplementary material for: Post-translational deregulation of YAP1 is genetically controlled in rat liver cancer and determines the fate and stem-like behavior of the human disease
Source: Oncotarget. 2016 Jun 23;7(31):49194–216. doi: 10.18632/oncotarget.10246 (PMC5226501; doi:10.18632/oncotarget.10246)
Supplement: Supplementary file 1 [file oncotarget-07-49194-s001.pdf]

Post-translational deregulation of YAP1 is genetically controlled in rat liver cancer and determines the fate and stem-like behavior of the human disease

Supplementary Materials

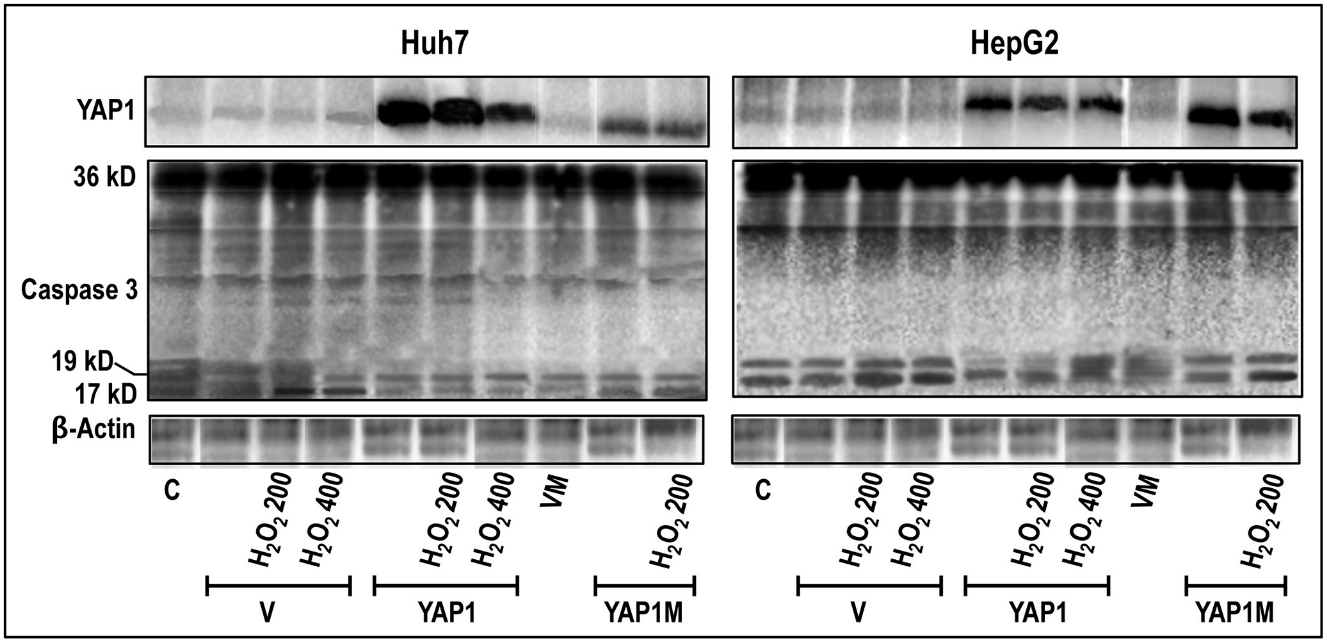

Supplementary Figure S1: Inhibition by YAP1 of apoptosis induced in Huh7 and HepG2 cells by oxygen peroxide. Representative Western blots of YAP1 and Caspase 3 cleavage. V, PCMV; VM, vector of mutated YAP1, YAP1M, mutated YAP1.

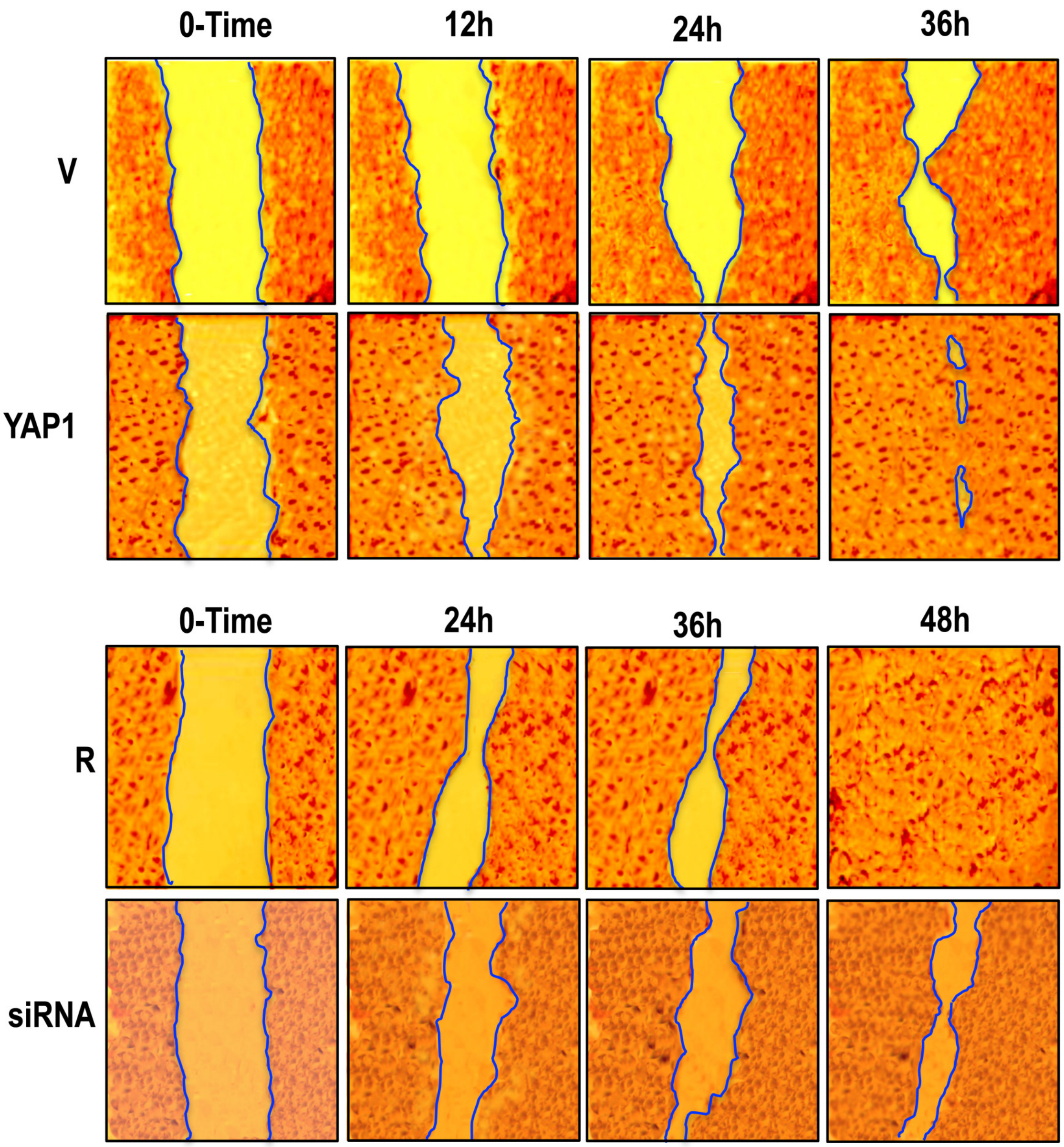

Supplementary Figure S2: The effect of YAP1 on Huh7 cell migration *in vitro*. Representative images of migration ability of cells transfected with YAP1 cDNA in pCMV6 vector or empty vector, V, (upper panels) or with siRNA anti-YAP1 or RNAiMAX, R, (lower panels), evaluted by wound healing assay. The restriction of the wounded area was evaluated at the times indicated after wounding (zero-time). Three independent analyses of cell migration *in vitro* did not show significant variations of the wounded area restriction at the different times.

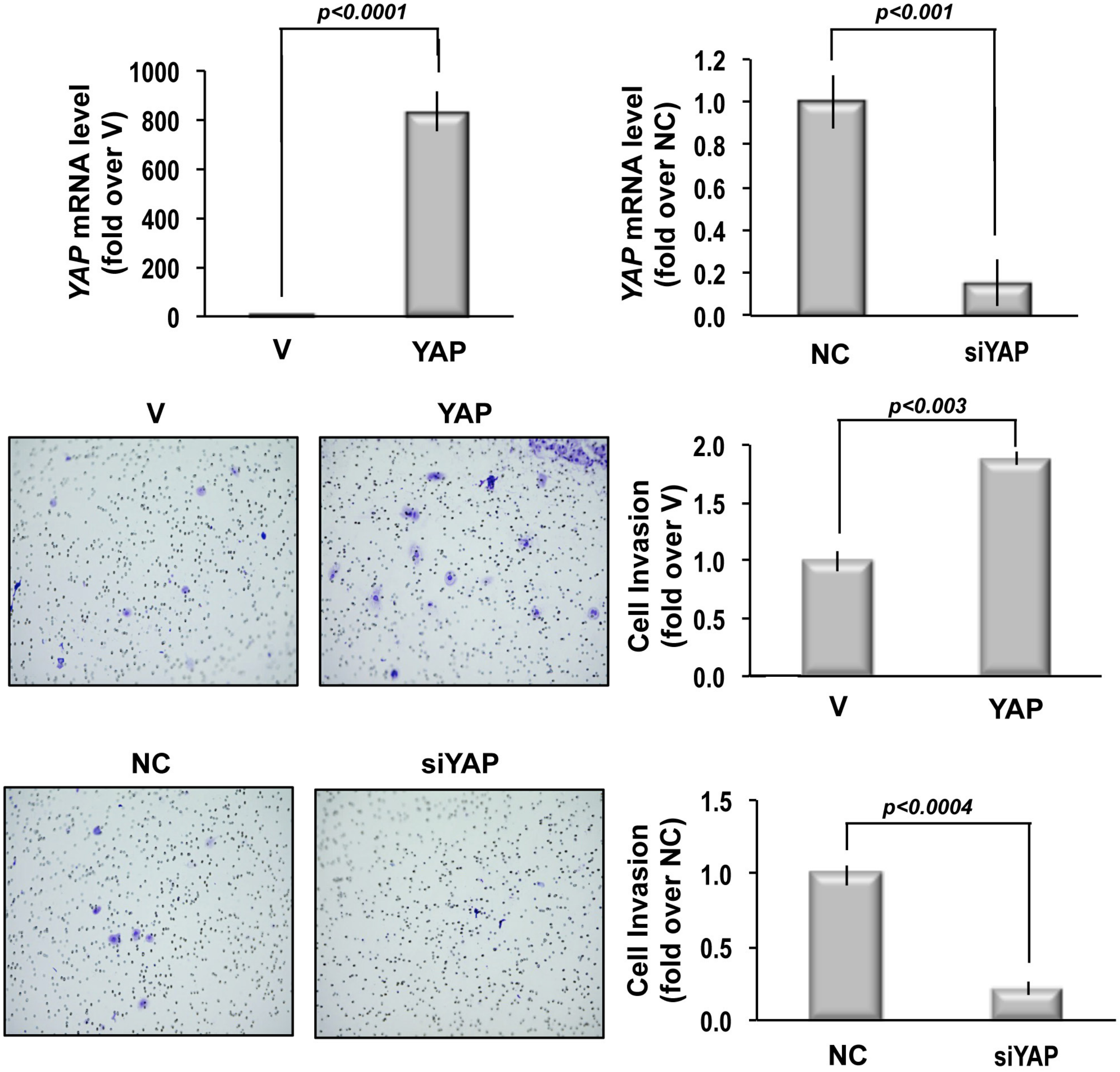

Supplementary Figure S3: Cell invasion assay. Huh7 cell invasivity was analyzed by the cell invasion assay kit with 50,000 cells/ well. Data are means (SD) of 3 experiments. Abbreviations: NC, negative control/scramble; V, vector.
